# Progerin impairs vascular smooth muscle cell growth *via* the DNA damage response pathway

**DOI:** 10.18632/oncotarget.15973

**Published:** 2017-03-07

**Authors:** Daisuke Kinoshita, Ayako Nagasawa, Ippei Shimizu, Takashi K. Ito, Yohko Yoshida, Masanori Tsuchida, Atsushi Iwama, Toshiya Hayano, Tohru Minamino

**Affiliations:** ^1^ Department of Cardiovascular Medicine, Chiba University Graduate School of Medicine, Chiba, Japan; ^2^ Department of Cardiovascular Biology and Medicine, Niigata University Graduate School of Medical and Dental Sciences, Niigata, Japan; ^3^ Department of Thoracic and Cardiovascular Surgery, Niigata University Graduate School of Medical and Dental Sciences, Niigata, Japan; ^4^ Division of Molecular Aging and Cell Biology, Niigata University Graduate School of Medical and Dental Sciences, Niigata, Japan; ^5^ Department of Cellular and Molecular Medicine, Graduate School of Medicine, Chiba University, Chiba, Japan; ^6^ Department of Biomedical Sciences, College of Life Sciences, Ritsumeikan University, Shiga, Japan

**Keywords:** cellular senescence, HGPS, DNA-PK, vascular smooth muscle cells, p53, Gerotarget

## Abstract

Mutations of the lamin A gene cause various premature aging syndromes, including Hutchinson-Gilford progeria syndrome (HGPS) and atypical Werner syndrome. In HGPS (but not atypical Werner syndrome), extensive loss of vascular smooth muscle cells leads to myocardial infarction with premature death. The underlying mechanisms how single gene mutations can cause various phenotypes are largely unknown. We performed an interactome analysis using mutant forms of lamin A involved in progeroid syndromes. We found that the mutant lamin A responsible for HGPS, known as progerin, could not bind to proteins related to the DNA damage response, including DNA-dependent protein kinase (DNA-PK). In contrast, wild-type lamin A and lamin A mutants causing atypical Werner syndrome were able to bind to these molecules. We also found that forced expression of progerin in vascular smooth muscle cells led to activation of DNA-PK and cellular growth arrest, while knockdown of DNA-PK attenuated this. Deletion of p53 also improved the inhibition of cell growth due to forced expression of progerin. These findings suggested that progerin activates the DNA damage response pathway and that dysregulation of this pathway may be responsible for the development of cardiovascular pathology in patients with HGPS.

## INTRODUCTION

Hutchinson-Gilford progeria syndrome (HGPS) is among the most severe premature aging disorders and patients with HGPS die of cardiovascular complications at an average age of only 13 years [1, 2]. While lifestyle-related atherosclerosis involves vascular endothelial cell dysfunction, endothelial cell function is normal in HGPS patients [1]. Instead, they show loss of vascular smooth muscle cells (VSMCs) from the arterial media, which causes maladaptive vascular remodeling and leads to myocardial infarction [1].

Lamin A is a component of the nuclear lamina, which lies on the inner surface of the nucleus, and it is involved in regulating multiple cellular functions such as maintenance of nuclear integrity, organization of chromatin, DNA replication, and transcription [3, 4]. Lamin A is encoded by the *LMNA* gene and mutations of this gene cause several diseases that develops through the degenerations of specific types of mesenchymal cells in muscle, white adipose tissue, and bone [3, 4]. Other *LMNA* mutations cause premature aging syndromes. The majority of HGPS patients have the G608G mutation that cause abnormal splicing in exon 11 to generate a truncated form of lamin A protein called progerin [3, 4]. In contrast, a subset of patients with Werner syndrome, a much less severe form of progeria with a median lifespan of 54 years [5], have missense mutations such as R133L and L140R [3, 4].

Several studies using mouse models or cells derived from progeria patients have suggested that DNA damage response pathways may be involved in the pathophysiology of HGPS. In Zmpste24^−/−^ mice that have similar phenotypic features to HGPS, the downstream targets of p53 were up-regulated and p53 deletion partially reversed some of the markers of premature aging including a shortened lifespan [6]. Phosphorylated histone H2AX is a marker of the response to DNA double-strand breaks, and it was found to be increased in these mice as well as in fibroblasts from HGPS patients [6, 7]. Ataxia telangiectasia mutated (ATM) is a kinase that is rapidly and specifically activated in response to DNA double-strand breaks, and it was found to be activated in Zmpste24^−/−^ mice as well as in Lmna G609G/G609G mice that produce progerin and display clinical features of HGPS [8]. Changes in the localization and expression of DNA-dependent protein kinase (DNA-PK), another DNA damage response kinase, have been reported in HGPS fibroblasts, although the role of DNA-PK in HGPS is not clear [9]. Studies using induced pluripotent stem cells obtained from HGPS patients have detected cell type-specific toxicity of progerin for VSMCs [9, 10], reflecting the unique pattern of arteriosclerosis in HGPS.

While there has been an increase in our knowledge of these syndromes, important questions remain unanswered. For example, “why do mutations of the same gene lead to such different premature aging phenotypes as HGPS and atypical Werner syndrome?”, “what causes cell-specific toxicity of progerin for cells with a mesenchymal origin?”, and “how are DNA damage response pathways related to the etiology of HGPS?”. In the present study, we attempted to address these issues by performing comparative interactome analysis of mutant forms of lamin A involved in HGPS and atypical Werner syndrome.

## RESULTS

### Interactome analysis of wild-type and mutant lamin A

**Figure 1:**
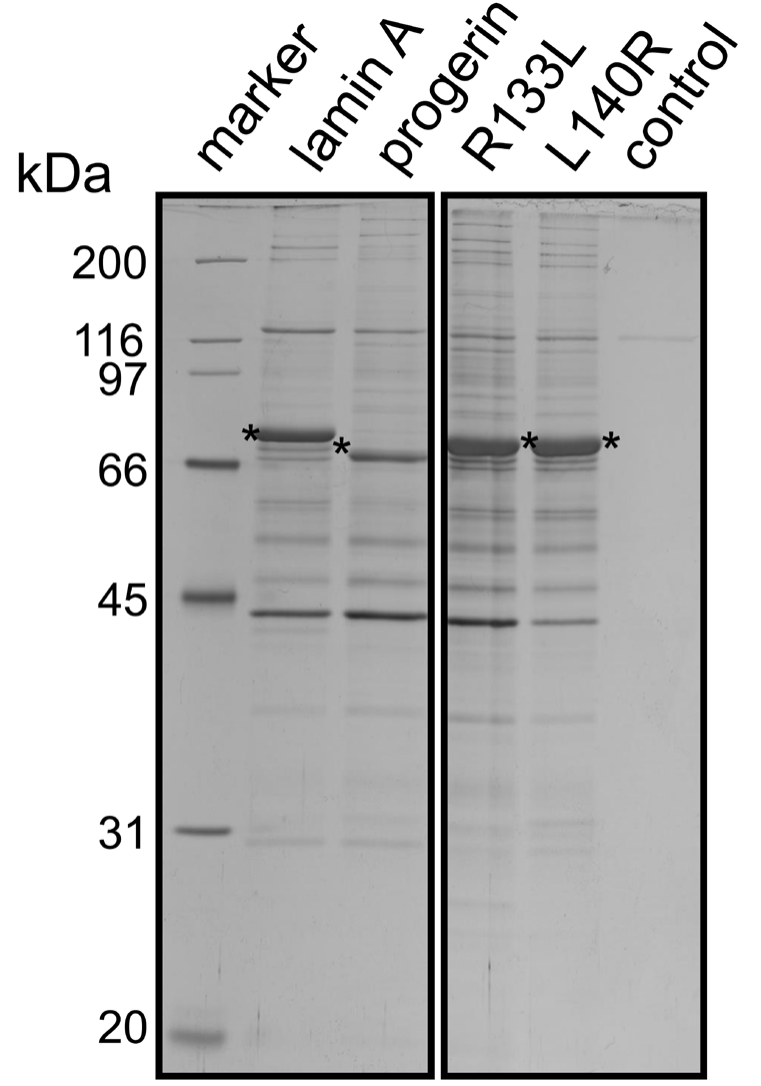
Isolation of lamin A or lamin A mutants-associated complexes Wild-type lamin A or lamin A mutants-associated complexes were isolated by immunoprecipitation from HEK293 cells transfected with lamin A or lamin A mutants expression vectors (progerin, R133L, L140R). The immune-isolated complexes were resolved by SDS-PAGE on a 12.5% gel followed by silver staining. Untransfected HEK 293 cells were used in control experiments (control). Asterisks indicate FLAG-tagged lamin A or lamin A mutants.

**Figure 2:**
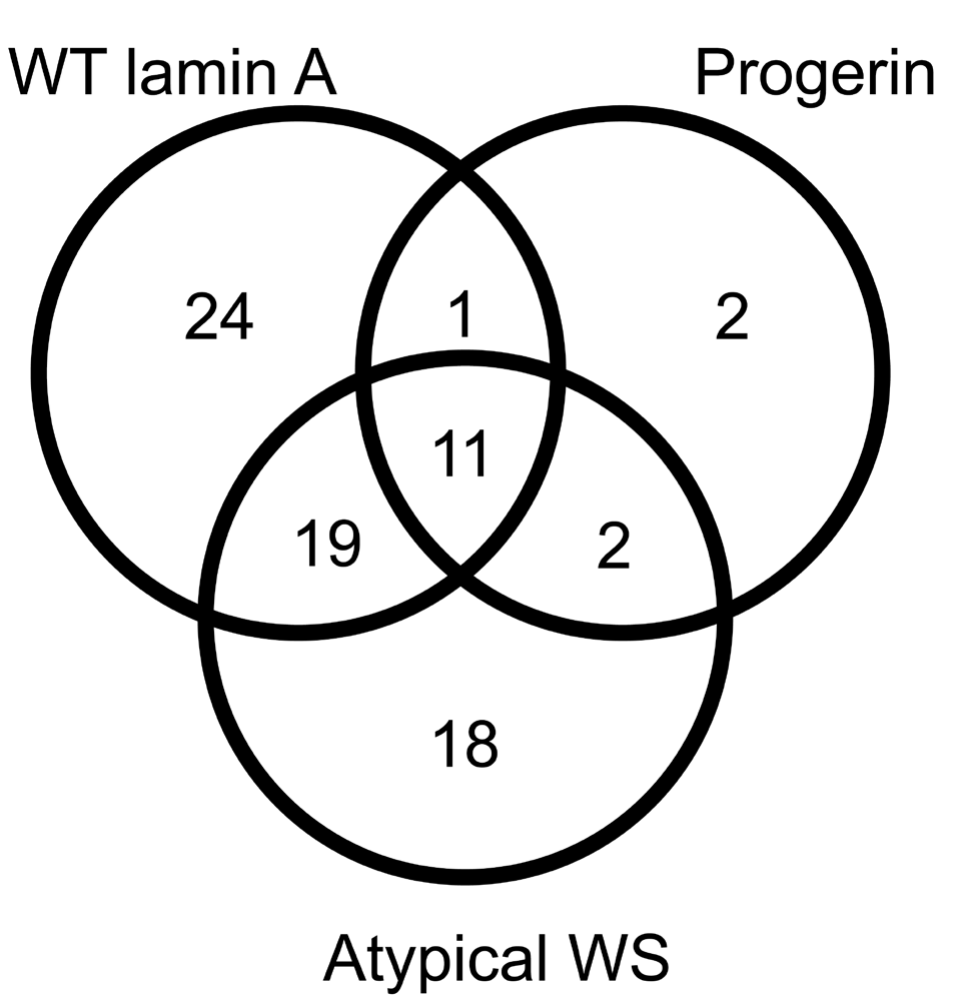
Venn diagram for the interactome analysis We identified 55 binding partners of wild-type lamin A, but most were not recognized by lamin A mutants, in particular, progerin.

In order to understand how mutation of lamin A causes more severe premature aging than other mutations in HGPS, we transfected HEK293 cells with four types of flag-tagged lamin A as the bait and performed immunoprecipitation of cell lysates with an anti-flag antibody. Binding proteins were subjected to LC-MS/MS analysis. The baits were flag-tagged wild-type lamin A, flag-tagged lamin A R133L (a mutant causing atypical Werner Syndrome), flag-tagged lamin A L140R (another mutant causing atypical Werner Syndrome), and flag-tagged progerin (Figure 1). We identified 55 binding partners of wild-type lamin A, which included some proteins previously reported to bind to lamin A, validating the quality of the present experiments (Supplemental Table 1). The three lamin A mutants retained the ability to bind to some of the proteins that bound to wild-type lamin A, but most were no longer recognized. L140R was unable to bind with 30 of the 55 proteins, while R133L lost the ability to bind to 32 proteins and progerin could not bind to 43 proteins (Figure 2 and Supplemental Table 2). We also found that the lamin A mutants could bind to a substantial number of proteins to which wild-type lamin A could not bind (Figure 2 and Supplemental Table 2). According to interactome analysis, progerin showed the largest loss and smallest gain in the number of protein-protein interactions compared with wild-type lamin A. These results led us to hypothesize that the normal lamin A protein network is most severely disrupted in HGPS among the laminopathies associated with premature aging and that alterations of protein interactions may contribute to the characteristic phenotypic features of this disease.

Next, we performed gene ontology (GO) analyses in order to detect functional differences between progerin-associated proteins. Using the functional annotation tool DAVID, we searched for GO terms concentrated in the sets of proteins binding to wild-type lamin A, R133L, L140R, or progerin. Because several GO terms with similar functions showed almost complete overlap, we manually categorized them into common biological terms (Table 1). We found seven terms that were significantly enriched in wild-type lamin A-bound proteins (Table 1). Interestingly, proteins that bound to any lamin A mutant causing atypical Werner syndrome usually shared almost all of these terms, although the interacting molecules did not correspond exactly to wild-type lamin A (Table 1 and Supplemental Table 2). It is noteworthy that progerin lacked two terms when compared with the other three proteins, which were “DNA damage response” and “muscle” (Table 1). These data suggest that progerin has lost a set of binding partners with specific functions rather than losing the ability to bind to proteins randomly.

**Table 1:** Gene ontology analyses for progerin-associated proteins

	lamin A	progerin	R133L	L140R
DNAdamage response	●		●	●
muscle	●			
cytoskeleton	●	●	●	●
intranuclear	●	●	●	●
nuclar lamina	●	●	●	●
nuclear membrane	●	●	●	●
nuclear periphery	●	●	●	●

Term	*P*-Value	laminA	progerin	R133L	L140R
**DNA Damage Response**					
DNA-dependent protein kinase-DNA ligase 4 complex	1.80E-02				
nonhomologous end joining complex	1.80E-02				
**muscle**					
contractile fiber	9.30E-03				
contractile fiber part	7.70E-03				
fascia adherens	3.50E-02				
I band	1.70E-02				
myofibril	7.40E-03				
sarcomere	5.20E-03				
Z disc	1.20E-02				
**Cytoskeleton**					
actin cytoskeleton	3.00E-08				
Arp2/3 protein complex	1.00E-11				
cortical actin cytoskeleton	9.60E-02				
cytoskeleton	2.10E-09				
cytoskeletal part	9.30E-08				
intermediate filament	4.90E-04				
intermediate filament cytoskeleton	5.40E-04				
lamin filament	1.30E-04				
spectrin	3.20E-02				
**intranuclear**					
chromatin	3.50E-02				
chromosome	3.10E-05				
chromosomal part	4.20E-04				
chromosome telomeric region	9.90E-02				
DNA replication factor A complex	2.10E-02				
intracellular organelle lumen	2.90E-04				
nuclear chromosome	2.70E-03				
nuclear chromosome part	9.50E-04				
nuclear chromosome telomeric region	7.30E-02				
nuclear matrix	6.60E-03				
nuclear replication fork	5.30E-02				
nuclear replisome	4.90E-02				
nuclear telomere cap complex	3.20E-02				
nucleoplasm	2.30E-04				
nucleoplasm part	4.80E-02				
replisome	4.90E-02				
telomere cap complex	3.20E-02				
**nuclear lamina**					
lamin filament	1.30E-04				
nuclear lamina	1.90E-04				
**nuclear membrane**					
endomembrane system	3.80E-04				
membrane-enclosed lumen	4.80E-04				
nuclear inner membrane	7.30E-05				
nuclear membrane	6.00E-06				
organelle membrane	9.50E-02				
organelle inner membrane	5.40E-03				
protein-DNA complex	2.50E-04				
**nuclear periphery**					
cell-cell adherens junction	8.90E-02				
cell cortex	9.70E-02				
cell leading edge	1.50E-03				
cell projection	3.80E-02				
envelope	1.50E-03				
intercalated disc	5.30E-02				
intracellular non-membrane-bounded organelle	4.60E-11				
intracellular organelle lumen	2.90E-04				
lamellipodium	2.60E-02				
membrane-enclosed lumen	4.80E-04				
non-membrane-bounded organelle	4.60E-11				
nuclear envelope	5.90E-07				
nuclear lumen	1.00E-04				
nuclear periphery	2.00E-02				
nuclear pore	3.30E-02				
organelle envelope	1.50E-03				
organelle lumen	3.80E-04				
pore complex	4.60E-02				

### Lack of progerin/DNA-PK interaction is associated with activation of DNA-PK

We assigned the three GO terms “DNA-dependent protein kinase (DNA-PK)-DNA ligase 4 complex”, “non-homologous end joining complex”, and “replication fork” to the category of DNA damage response (Table 1). DNA-PKcs is the catalytic subunit of DNA-PK, a nuclear DNA-dependent serine/threonine protein kinase [11]. DNA-PKcs is required for the non-homologous end joining (NHEJ) DNA repair pathway, which repairs double-strand breaks in DNA. We focused on the term “DNA-dependent protein kinase-DNA ligase 4 complex” because DNA-PKcs and its binding partners Ku80 and Ku70 were all detected in the interactomes of wild-type lamin A, R133L, and L140R, while none of them were detected among the progerin-associated proteins (Supplemental Table 2).

**Figure 3:**
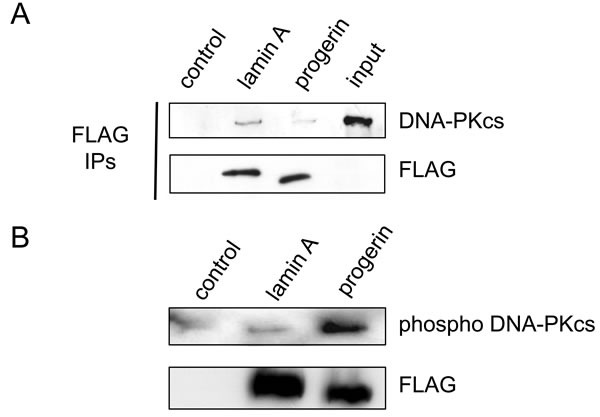
Progerin loses affinity with DNA-PK and activates it **A**. An expression vector encoding flag-tagged wild-type lamin A or progerin was transduced into HEK293 cells, after which co-immunoprecipitation was conducted with anti-flag antibody. Then the samples were subjected to western blot analysis for detection of DNA-PKcs and flag protein. The empty vector was used as a control. **B**. An expression vector encoding flag-tagged wild-type lamin A or progerin was transduced into HEK293 cells, following which the levels of phosphorylated DNA-PKcs and flag protein were examined by western blot analysis.

Many proteins have been identified as substrates for the kinase activity of DNA-PK. Autophosphorylation of DNA-PKcs appears to activate the NHEJ pathway of DNA repair and is thought to induce a conformational change that allows end-processing enzymes to access the ends of the double-strand break. In addition, DNA-PKcs has been reported to bind to a number of regulators of DNA metabolism. To investigate the interaction between progerin and DNA-PKcs, we transfected cells with flag-tagged lamin A or flag-tagged progerin and then performed immunoprecipitation with an anti-flag antibody and western blot analysis for DNA-PKcs expression. DNA-PKcs was easily detected when cells were transfected with flag-tagged lamin A, while it was markedly reduced in cells transfected with flag-tagged progerin (Figure 3A), validating the results obtained by mass spectrometry. To examine how lamin A influenced the activation of DNA-PKcs, we further assessed its phosphorylation. While introduction of wild-type lamin A did not up-regulate the phosphorylation of DNA-PKcs, introduction of progerin led to a marked increase of its phosphorylation (Figure 3B). These results suggested that accumulation of progerin could increase the activity of DNA-PK by changing its binding properties.

### Progerin impairs VSMC growth in a DNA-PK and p53-dependent manner

**Figure 4:**
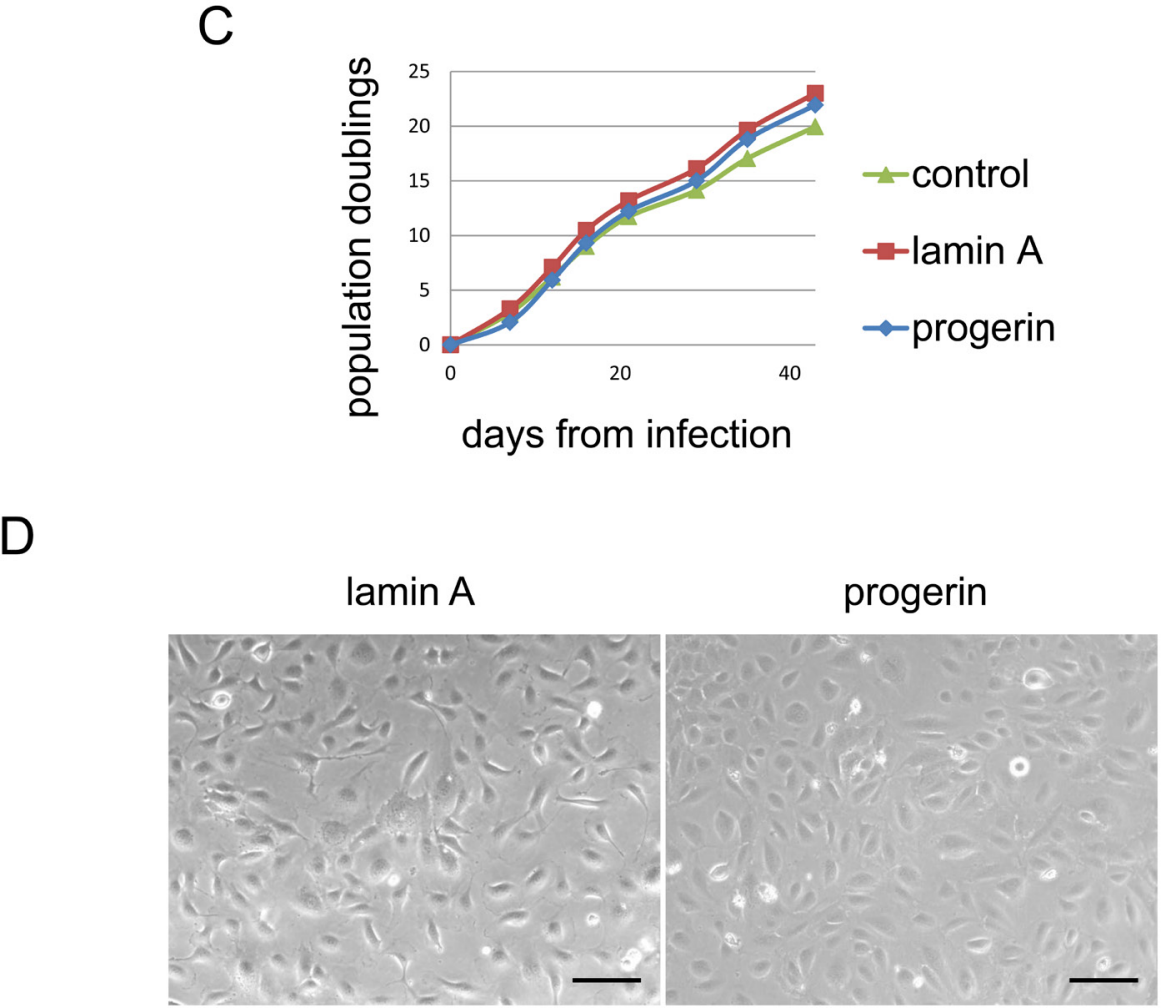
Progerin impairs the growth of VSMCs but not HUVECs **A**. VSMCs were infected with a retroviral vector encoding wild-type lamin A or progerin and population doubling was examined. **B**. Representative pictures of VSMCs infected with the wild-type lamin A vector or progerin vector. Scale bar = 100 μm. **C**. HUVECs were infected with a retroviral vector encoding wild-type lamin A or progerin and population doubling was examined. **D**. Representative pictures of HUVECs infected with the wild-type lamin A vector or the progerin vector. Scale bar = 100 μm.

Because VSMCs rather than vascular endothelial cells are involved in arteriosclerosis associated with HGPS, we tested the influence of progerin on the growth of VSMCs and human umbilical vein endothelial cells (HUVECs) by using a retroviral vector that encoded wild-type lamin A or progerin. Introduction of progerin into VSMCs strongly reduced cell growth and shortened the replicative lifespan (Figure 4A, 4B and Supplemental Figure 1A). In contrast, introduction of progerin had no effect on the growth and lifespan of HUVECs (Figure 4C, 4D and Supplemental Figure 1B), showing a cell type-specific effect of progerin. These results are consistent with the known pathology of HGPS as well as with a previous report about iPS cells [10]. We found that expression of apoptosis markers such as *BAX, PMAIP1(NOXA)* and *BBC3(PUMA)* was markedly reduced in progerin-infected VSMCs (Supplemental Figure 1C), suggesting that apoptotic cell death may have a minor role for the phenotype observed in our model.

**Figure 5:**
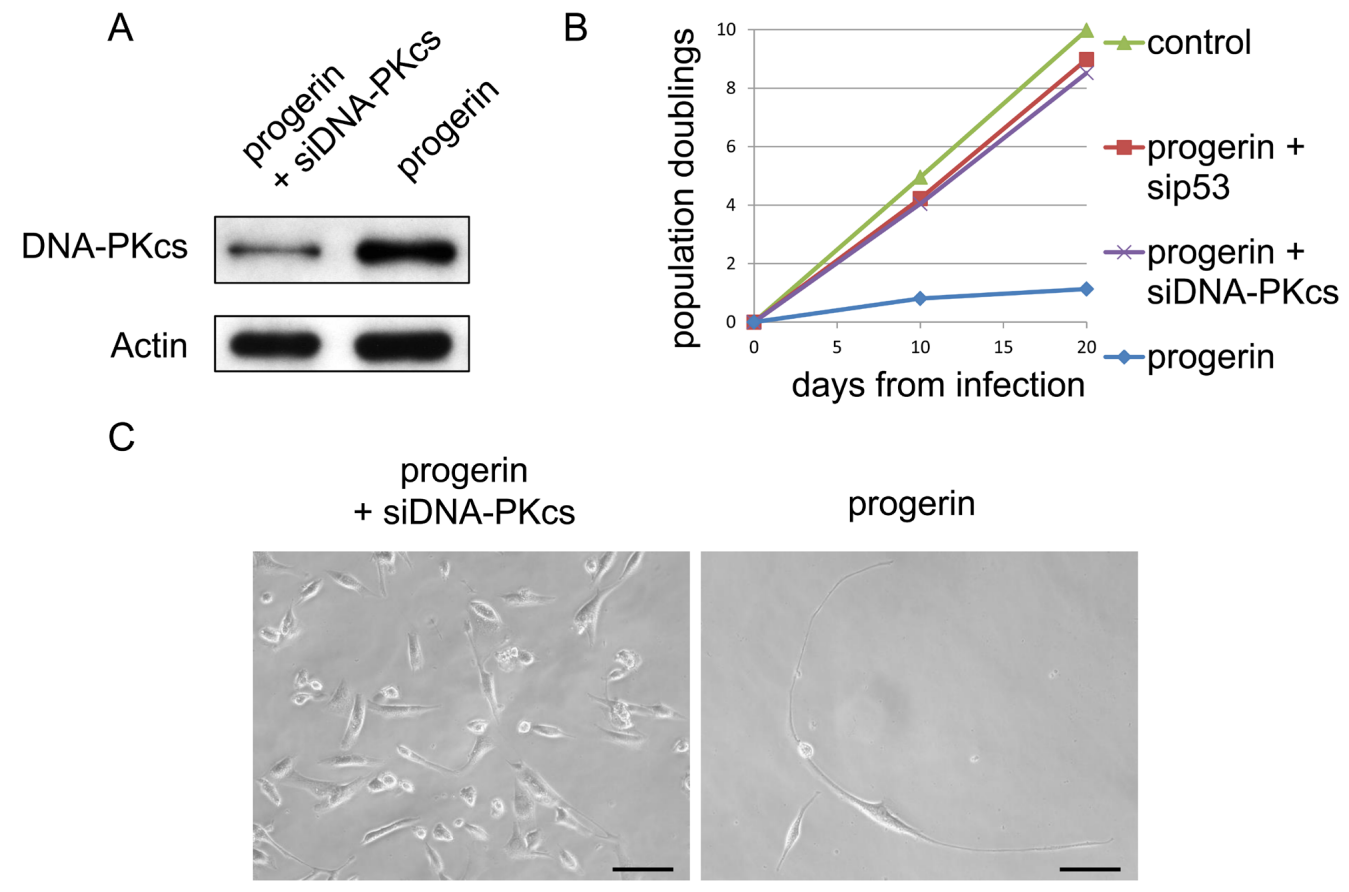
DNA-PKcs knockdown attenuates progerin-induced inhibition of VSMC growth **A**. VSMCs were infected with a retroviral vector encoding progerin with or without siRNA targeting DNA-PKcs, after which expression of DNA-PKcs was examined by western blot analysis. **B**. Population doubling was examined using VSMCs prepared as explained in Figure 5A. In some groups, VSMCs were infected with a retroviral vector encoding progerin with or without siRNA targeting p53, after which population doubling was examined. **C**. Representative pictures of VSMCs prepared as explained in (A). Scale bar = 100 μm.

We next examined whether knockdown of DNA-PKcs with small interfering RNA (siRNA) could improve the growth of VSMCs after introduction of progerin. We found that siRNA targeting DNA-PKcs reduced the DNA-PKcs protein level in cultured VSMCs (Figure 5A). In addition, knockdown of DNA-PKcs reversed the suppression of cell growth by progerin (Figure 5B and 5C), suggesting that activation of DNA-PK could account for the anti-proliferative effect of progerin on VSMCs.

**Figure 6:**
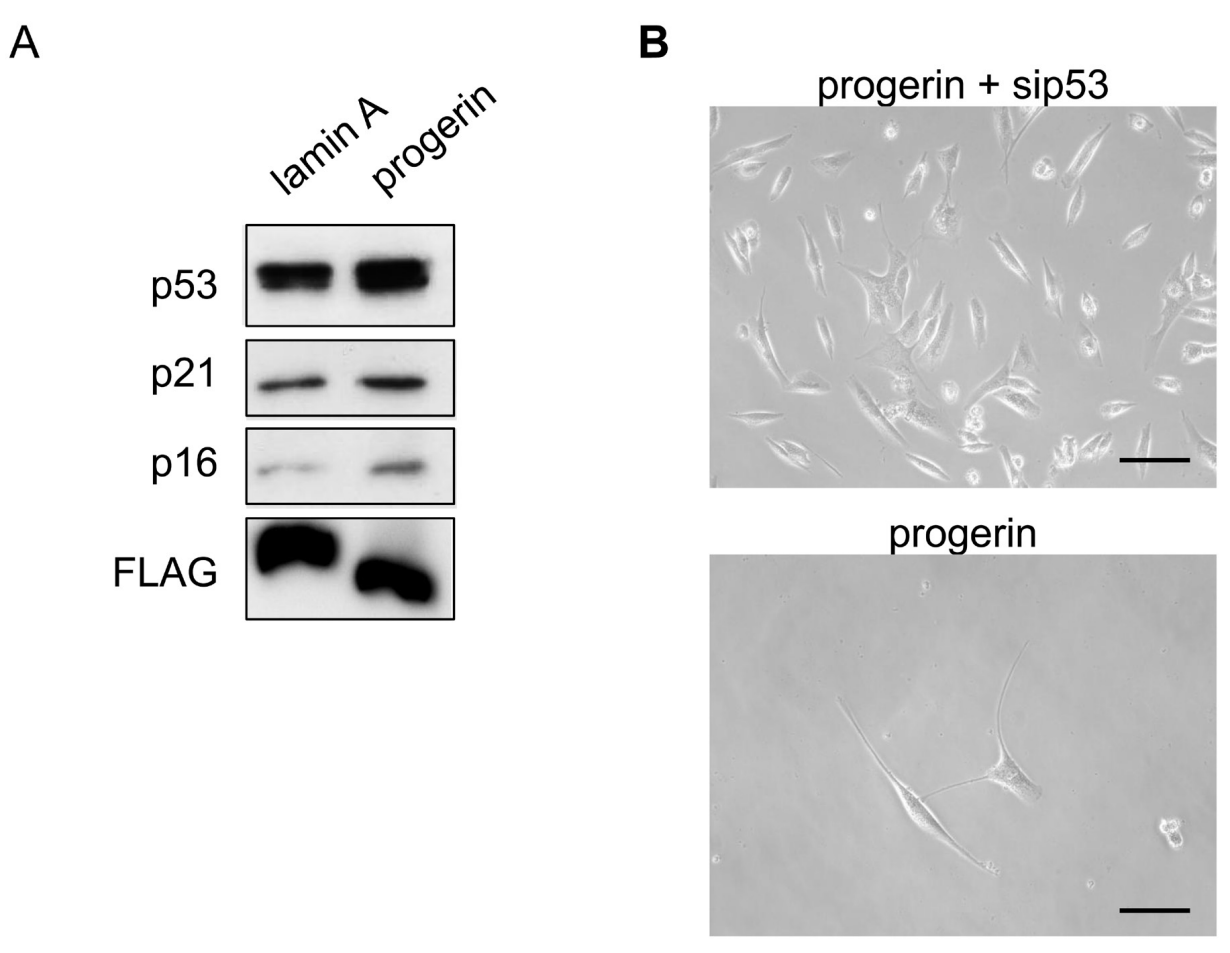
p53 knockdown improves progerin-induced inhibition of VSMC growth **A**. VSMCs were infected with a retroviral vector encoding wild-type lamin A or progerin, following which expression of p53, p21, and p16 was examined by western blot analysis. **B**. Representative pictures of VSMCs prepared as explained in Figure 5B. Scale bar = 100 μm.

VSMC numbers did not increase when culture was continued for 2 months after introduction of progerin. When we harvested these cells and performed western blot analysis of cell cycle checkpoint proteins related to the DNA damage response and cellular senescence, we found that expression of p53, p21, and p16 was increased by introduction of progerin compared with wild-type lamin A (Figure 6A). It is well known that DNA-PK cooperates with ATM, leading to phosphorylation and activation of p53 [12, 13]. Accordingly, we speculated that progerin-induced activation of DNA-PK might up-regulate p53 activity, thereby inducing cell growth arrest. To test this hypothesis, we introduced siRNA targeting p53 into progerin-infected VSMCs and found that this siRNA counteracted the anti-proliferative effect of progerin on VSMC growth (Figure 5B and 6B). These results suggested that progerin suppresses VSMC growth via activation of the DNA damage response pathway.

### Progerin upregulates pro-inflammatory gene expression in a NF-κB-dependent, but DNA-PK-independent, manner

**Figure 7:**
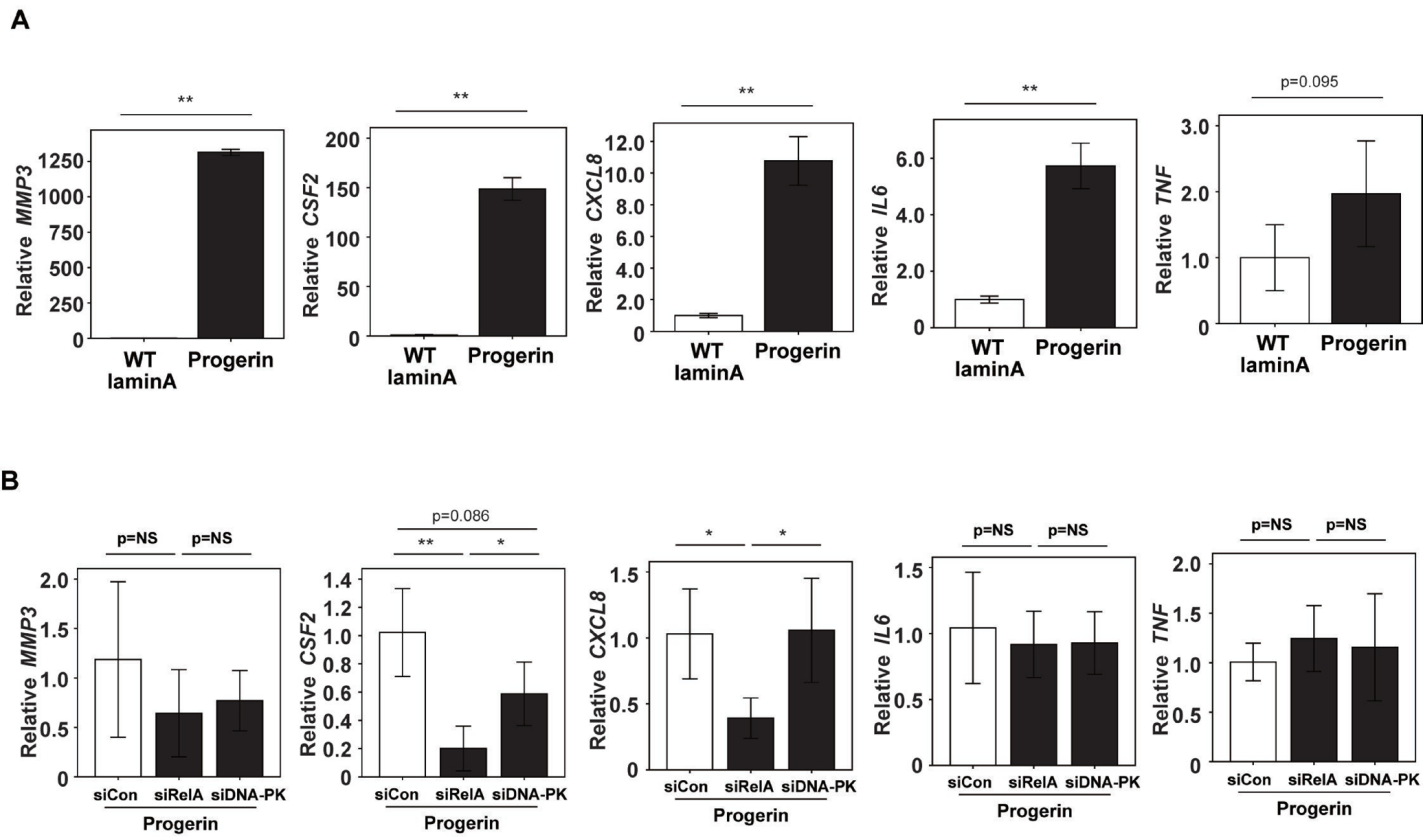
Knockdown of RelA, but not DNA-PK, attenuates progerin-induced upregulation of pro-inflammatory molecules in VSMCs **A**. VSMCs were infected with a retroviral vector encoding wild-type lamin A or progerin. Then expression of *MMP3*, *CSF2*, *CXCL8*, *IL6* and *TNF* was examined by micro array analysis. Control siRNA was also transfected as a control (*n* = 4,4). **B**. VSMCs were infected with a retroviral vector encoding progerin and with siRNA targeting the NF-κB component RELA (p65) or DNA-PKcs. Then expression of *MMP3*, *CSF2*, *CXCL8*, *IL6* and *TNF* was examined by real time PCR (*n* = 3,3). NS, not significant. * *P* < 0.05. ** *P* < 0.01. Data are shown as the mean ± SEM. Differences between groups were examined by 2-tailed Student's t-test.

Accumulation of prelamin A isoforms at the nuclear lamina was reported to trigger activation of NF-κB and secretion of high levels of pro-inflammatory cytokines in two different mouse models of HGPS [8]. We investigated whether progerin overexpression in VSMCs up-regulated pro-inflammatory molecules via a DNA-PK-dependent pathway. Microarray analysis revealed the up-regulation of pro-inflammatory cytokines production and extracellular proteases (including *MMP-3, CSF2, CXCL8, IL6* and *TNF*) by VSMCs after introduction of progerin compared with wild-type lamin A (Figure 7A and Supplemental Table 3). Inhibition of NF-κB signaling by siRNA targeting *RELA* (p65) attenuated the progerin-induced upregulation of *CSF2* and *CXCL8*, while *MMP-3, IL6* and *TNF* expression were similar between the groups (Figure 7B). We next tested three siRNAs targeting DNA-PKcs or ATM, but none of them downregulated the expression of these pro-inflammatory molecules (Figure 7B and data not shown), suggesting that progerin-induced inflammation is independent of DNA-PK activation.

## DISCUSSION

In the present study, we performed affinity purification using wild-type lamin A and three mutant forms of lamin A associated with premature aging syndromes as the bait, employing mass spectrometry for analysis of the samples. As a result, we identified over 50 proteins that had not been previously recognized as binding to lamin A. We found that part of this protein network was shared between wild-type lamin A and the lamin A mutants causing atypical Werner syndrome, although more than half of these proteins did not bind to the mutants. In addition, only a few of the proteins bound to progerin, suggesting that the mutation underlying HGPS most severely impairs the ability of lamin A to bind to functional proteins. Consistent with this concept, our GO functional analysis revealed that progerin did not bind to a set of proteins that had functions categorized into “DNA damage response” and “muscle”, while the two lamin A mutants causing atypical Werner syndrome still bound to proteins related to the “DNA damage response”. Since about 25% of lamin A-binding proteins fitted into this category, we focused on the DNA damage response. Among DNA-PKcs and its associated proteins, Ku80 and Ku70 were found to bind to wild-type lamin A and the two mutants causing atypical Werner syndrome, but not with progerin. We also demonstrated that overexpression of progerin upregulated DNA-PKcs phosphorylation in VSMCs and induced premature senescence of these cells, while DNA-PK knockdown reversed progerin-induced cell growth arrest, providing evidence that DNA-PK could contribute to the pathogenesis of HGPS.

While data from several mouse models of HGPS have suggested the importance of the DNA damage response [6, 8, 9], the actual role of this pathway remains unclear. Osorio et al. reported that accumulation of progerin triggers a signaling pathway involving ATM that activates NF-κB [8], and they also demonstrated that NF-κB-driven inflammation is responsible for several important features of the progeroid phenotypes. Interestingly, inhibition of the NF-κB pathway by genetic or pharmacological strategies was able to prevent these phenotypic alterations, demonstrating the causal role of this inflammatory pathway in the pathogenesis of accelerated aging. In agreement with their results, we showed that introduction of progerin into VSMCs led to upregulation of pro-inflammatory molecules, while inhibition of NF-κB signaling attenuated the inflammatory response induced by progerin. However, inhibition of ATM or DNA-PK failed to inhibit progerin-induced upregulation of pro-inflammatory molecules in VSMCs, suggesting that progerin induces inflammation via a mechanism that is independent of the DNA damage response.

Signaling kinases (ATM, ATR and, DNA-PK) are recruited to sites of DNA damage and are activated at these sites, leading to the local assembly of checkpoint and DNA repair factors and promoting the phosphorylation of transducer kinases (Chk1 and Chk2), which converge on p53 [14]. Marked upregulation of p53 target genes has been reported in a mouse model of HGPS, while blocking the activation of p53 attenuates premature aging [6]. In aged HGPS cells, Liu et al. found an increase of phosphorylated Chk1 and Chk2 owing to the activation of ATM and ATR [15]. Phosphorylated p53 was also increased, demonstrating that the ATR and ATM checkpoints were persistently activated, as also confirmed by other researchers [16–18]. The persistent activation of ATM/ATR in HGPS is thought to reflect a delay in DNA repair efficiency in these cells [15]; however, it was largely unknown how progerin impairs the DNA repair system. Recently, several lines of evidence suggest that lamins A maintain a nucleoplasmic pool of molecules related to the DNA repair system in order to facilitate its rapid recruitment to sites of DNA damage [19–21]. The inability of progerin to bind to these molecules could cause impairment of the DNA repair system, leading to the sustained activation of DNA damage response pathway [19–21], which suggests that progerin-induced phosphorylation of DNA-PKcs also reflects the impaired DNA repair system in HGPS cells.

Various explanations have been proposed for the cell type-specific pathology of laminopathies, including progerin-mediated exhaustion of stem cell pools [22], defects of mesenchymal lineage differentiation [23], impairment of the DNA damage repair response [24], and nuclear fragility in mechanically stressed cells such as cardiomyocytes [25]. Recently, Liu et al. generated induced pluripotent stem cells (iPSCs) from the fibroblasts of patients with HGPS. They elegantly demonstrated that progerin expression and associated premature senescence were markedly suppressed in the pluripotent state, while the aging phenotype emerged along with progerin expression after differentiation of the cells into VSMCs. However, this does not occur when HGPS-iPSCs undergo differentiation into fibroblasts [9]. Zhang et al. also generated HGPS-iPSCs and found that progerin levels were highest in mesenchymal stem cells (MSCs) and VSMCs differentiated from the pluripotent cells [10]. Because these cells were sensitive to various stresses, they speculated that progeria may be related to a shortage of MSCs needed for tissue repair. In the present study, we observed that forced expression of progerin induced premature senescence of VSMCs, but not endothelial cells, although the mechanisms underlying these observations are still obscure.

In conclusion, our findings suggest that progerin inhibits VSMC growth via the DNA damage response pathway and that this pathway probably contributes to the pathogenesis of HGPS. Further interactome analysis of lamin A mutants involved in premature aging syndromes could provide a platform for future studies on the link between lamin A and premature aging.

## MATERIALS AND METHODS

### Cell culture

HEK293 cells were cultured in DMEM (Sigma) supplemented with 10% FBS, streptomycin (0.1 μg ml^−1^), and penicillin G (100 units ml^−1^). Human VSMCs and HUVECs were purchased from Lonza and cultured according to the manufacturer's instructions. Culture was done at 37°C in a humidified incubator with 5% CO_2_ and proliferation was assessed by counting cells after subculture. We defined senescent cells as those that did not increase in number and remained subconfluent after 2 weeks of culture. The number of population doublings (PD) was calculated as follows: PD = log (number of cells after culture/initial number of cells)/log 2.

### Transfection of expression vectors and retroviral infection

The expression vector pcDNA™5/FRT/TO (Invitrogen) was cut with *Bam* H I and *Xho* I. Fragments of lamin A, progerin, lamin A R133L, or lamin A L140R were ligated into the corresponding restriction sites of pcDNA™5/FRT/TO. The resulting expression vectors were transfected into HEK293 cells by using Fugene. We created vectors based on pLNCX2 (Clontech, Palo Alto, CA, USA) that expressed wild-type lamin A or progerin for retroviral infection, which was done as described previously [26]. Briefly, VSMCs or HUVECs (passages 4-6) were plated at 5×10^5^ cells in 100-mm dishes at 24 hours before infection. Then the culture medium was replaced by retroviral stock medium supplemented with 8 μg ml^−1^ polybrene (Sigma). From 48 hours after infection, the cells were selected by culture for 7 days in 500 μg ml^−1^ G418. After selection, 2×10^5^ cells were seeded in 100-mm dishes on the 8th day post-infection, which was designated as day 0. The respective empty vectors were used as controls. In some experiments, after retroviral infection had been performed, siRNAs purchased from Ambion or Invitrogen were transfected at 10 nmol l^−1^ with RNAiFect (Qiagen) or Lipofectamine RNAiMAX (Invitrogen) according to the instructions of the respective manufacturers.

### Western blot analysis

Whole-cell lysates were prepared in lysis buffer (10 mM Tris-HCl, pH 8, 140 mM NaCl, 5 mM EDTA, 0.025% NaN3, 1% Triton X-100, 1% deoxycholate, 0.1% SDS, 1 mM PMSF, 5 µg ml^−1^ leupeptin, 2 µg ml^−1^ aprotinin, 50 mM NaF, and 1 mM Na_2_VO_3_). The lysates (40-50 µg) were resolved by SDS-PAGE and proteins were transferred to a PVDF membrane (Millipore), which was incubated with the primary antibody followed by anti-rabbit or anti-mouse immunoglobulin-G conjugated with horseradish peroxidase (Jackson). Specific proteins were detected by the enhanced chemiluminescence method (Amersham). The primary antibodies used for western blotting were as follows: anti-p53 antibody (#2524), anti-actin antibody (#4967) (Cell Signaling), and anti-GAPDH antibody (sc-20357, Santa Cruz), anti-DNA-PK cs antibody (sc-9051, Santa Cruz), anti-phospho-DNA-PK cs antibody (Thr2609) (sc-101664, Santa Cruz), anti-Rabbit Monoclonal Anti-FLAG antibody (F2555, Sigma).

### Real-time PCR

Total RNA (1 µg) was isolated from tissue samples with RNA-Bee (Tel-Test Inc.). Real-time PCR (qPCR) was performed by using a Light Cycler 480 (Roche) with the Taqman Universal Probe Library and the Light Cycler 480 Probes Master (Roche) according to the manufacturer's instructions. For quantification of the copy number, we used Light Cycler 480 software (version 1.5, Roche) and employed the ‘fit points’ method according to the manufacturer's instructions. GAPDH was used to normalize the amount of mRNA in each sample.

### Transcriptome analysis

VSMCs with p53 deficiency (overexpressing E6) were infected with retroviral vectors encoding lamin A or progerin and cells were harvested after 30 days. Then total RNA was isolated with RNA-Bee (Tel-Test, Inc.) and the genetic profile of lamin A and progerin were analyzed by using a Human Gene Expression 4×44K v2 Microarray Kit (Agilent Technologies) (*n* = 4 per group). Raw data were subjected to log2 transformation and normalized by using GeneSpring GX v7.3.1 (Agilent Technologies). Genes showing differential expression (*q* < 0.05) were determined by BH-FDR. Gene ontology analysis was performed using the Database for Annotation, Visualization and Integrated Discovery (DAVID) v6.7. Finally, the gene expression data thus obtained were deposited in the Gene Expression Omnibus database (GSE47553).

### Identification of proteins by LC-MS/MS

Immunoprecipitated protein samples (approximately 100 μl) were mixed with 400 μl of methanol. 100 μl chloroform was mixed with the samples, followed by the addition of 300 μl distilled water and stirred for 2 minutes. After centrifugation at 2,000 rpm for 2 minutes, the supernatant was removed and 300 μl methanol was added. After centrifugation at 15,000 rpm for 5 minutes, the supernatant was removed and the samples were dried with an evaporator. Dried samples were dissolved with the buffer including 180 mg urea, 0.12 M Tris HCl buffer 1.5M Tris HCl (pH 8.8) 33.3 μl, and H_2_O 370 μl, then mixed with 2 μl Tris (2-CarboxyEthyl) Phosphine (TCEP), and incubated at 37°C for 2 hours. One μl of methyl-methane-thio-sulphonate (MMTS) was added and incubated at room temperature for 10 minutes, followed by overnight incubation with 1 μl lysyl endopeptidase. Digested samples were dried with an evaporator, and then dissolved with 200 μl of 2% acetonitrile/0.1% trifluoroacetic acid. The samples were desalted with a tip made with C18 Empore Disk. The tips including samples were added with 100 μl of 70% acetonitrile/0.1% trifluoroacetic acid. Samples were centrifuged at 2,000 rpm for 5 minutes and the flow-through was discarded. 100 μl of 2% acetonitrile/0.1% trifluoroacetic acid was added and centrifuged at 2,000 rpm for 5 minutes. The flow-through was discarded followed by the addition of 200 μl of 2% acetonitrile/0.1% trifluoroacetic acid. Each 100 μl of samples was added to 2 tips, and was centrifuged at 2,000 rpm for 5 minutes, followed by the discard of the flow-through. The tip was loaded onto a new 1.5 ml tube, 100 μl of 2% acetonitrile/0.1% trifluoroacetic acid was added onto the tip, and the tubes were centrifuged. Eluted samples were dried with an evaporator, and dissolved with 12 μl of 2% acetonitrile/0.1% formic acid, followed by centrifugation at 15,000 rpm for 5 minutes. The supernatant was used for LC-MS/MS analysis. The data were analyzed using MASCOT software (Matrix Science, Wyndham Place, UK). Biogrid (http://thebiogrid.org/) was used to analyze if identified proteins are novel or previously reported. The proteins thus identified were categorized with the Database for Annotation, Visualization and Integrated Discovery (DAVID v6.7).

### Statistical analysis

Results are shown as the mean ± SEM. The two-tailed Student's *t*-test or one-way ANOVA was used to assess the statistical significance of differences. In all analyses, *P* < 0.05 was considered significant.

## SUPPLEMENTARY MATERIALS FIGURES AND TABLES








