# Supplementary material for: Progerin impairs vascular smooth muscle cell growth via the DNA damage response pathway
Source: Oncotarget. 2017 Mar 7;8(21):34045–56. doi: 10.18632/oncotarget.15973 (PMC5470950; doi:10.18632/oncotarget.15973)
Supplement: Supplementary file 1 [file oncotarget-08-34045-s001.pdf]

Progerin impairs vascular smooth muscle cell growth *via* the DNA damage response pathway

Supplementary Material

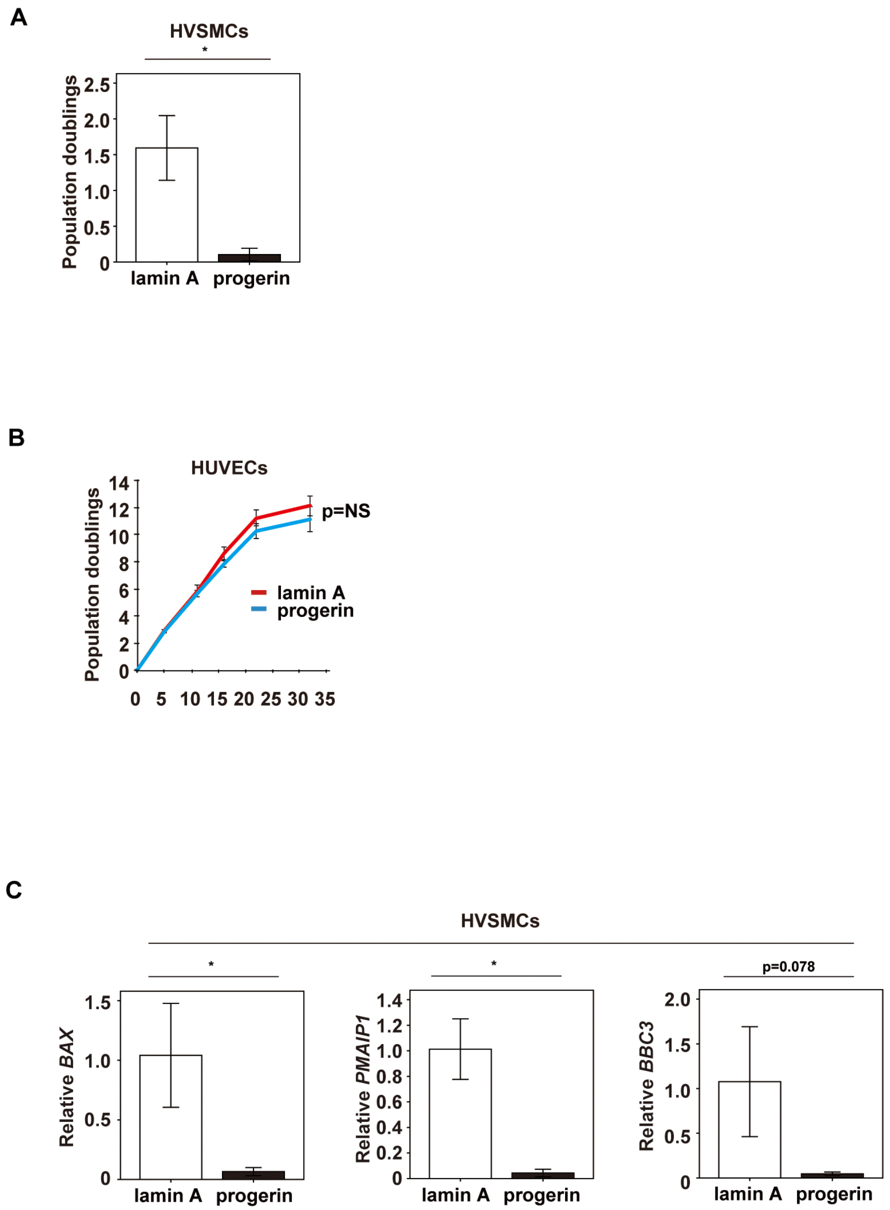

Supplemental Figure 1: Progerin impairs the growth of VSMCs via a p53 and/or DNA-PK mediated signaling (A) In another series of the experiments, reproducibility was analyzed. VSMCs were infected with a retroviral vector encoding wild-type lamin A or progerin and population doubling was examined at day 9 after the termination of cell selection (n=3,3). (B) To confirm reproducibility, another series of the experiments were done with HUVECs infected with a retroviral vector encoding wild-type lamin A or progerin, and population doubling was examined. The p-value was evaluated to be non-significant (p=NS) with non-parametric Kruskal Wallis test (n=3,3). (C) VSMCs were infected with a retroviral vector encoding wild-type lamin A or progerin. Forty five days after the termination of cell selection, expression levels of *BAX, PMAIP1(NOXA)* and *BBC3(PUMA)* were examined by a real time PCR (n=3,3). * P < 0.05. Data are shown as the mean ± SEM. Differences between groups were examined by 2-tailed Student's *t*-test.
