# Supplementary figures and images for: Progerin impairs vascular smooth muscle cell growth via the DNA damage response pathway
Source: Oncotarget. 2017 Mar 7;8(21):34045–56. doi: 10.18632/oncotarget.15973 (PMC5470950; doi:10.18632/oncotarget.15973)

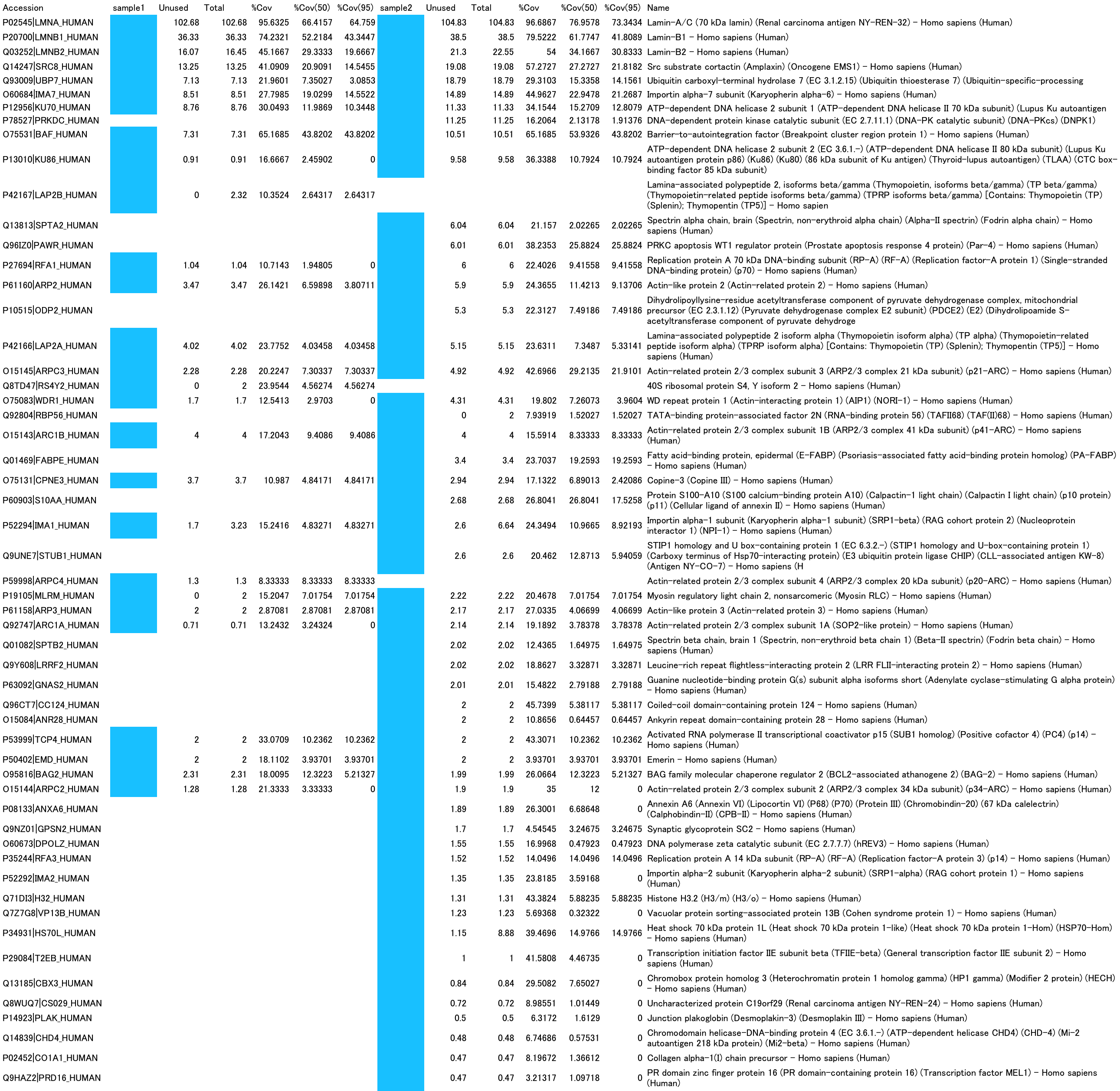

Supplement: Supplementary file 2 [file oncotarget-08-34045-s002.docx]

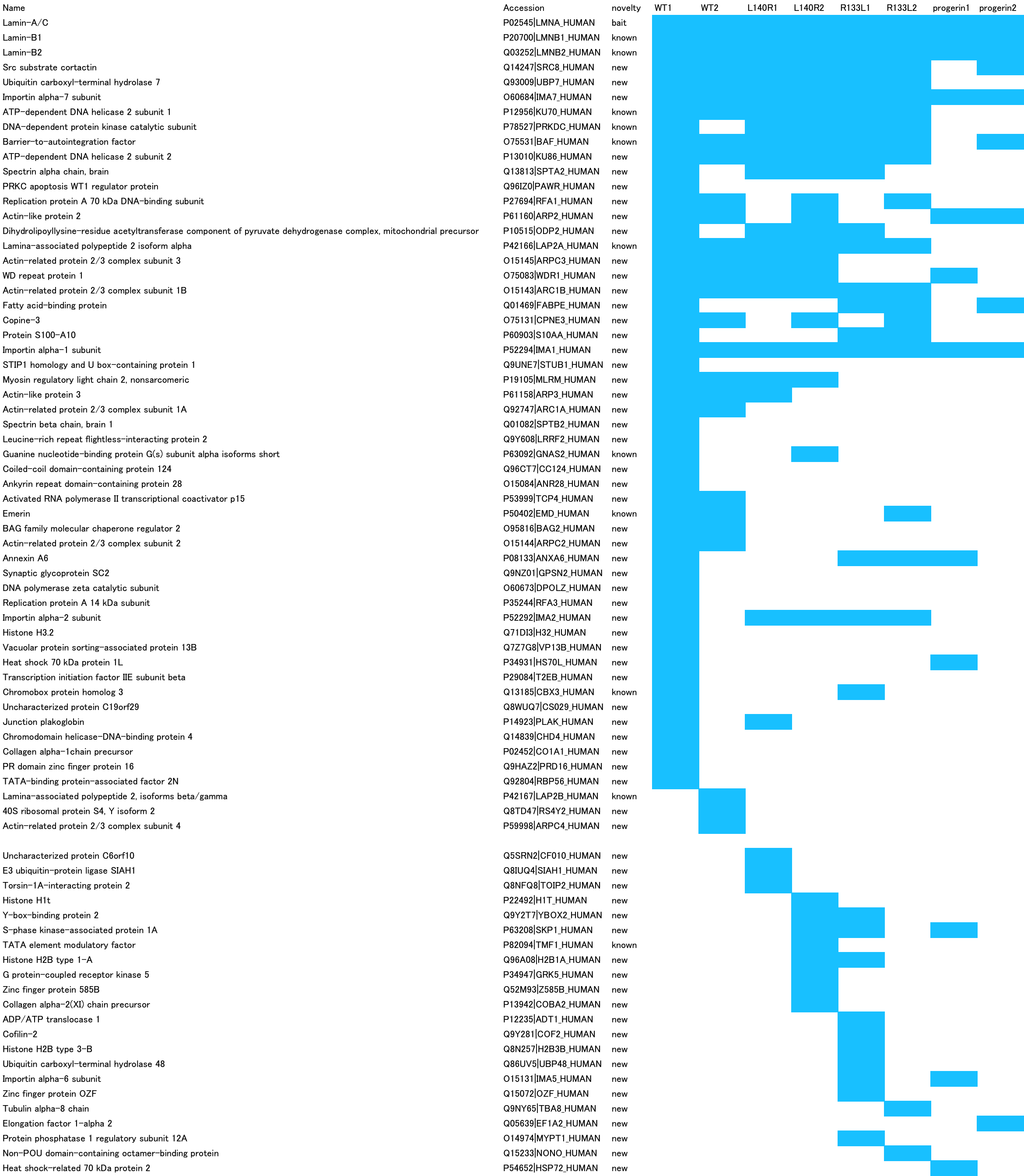

Supplement: Supplementary file 3 [file oncotarget-08-34045-s003.docx]
