# Supplementary material for: Progerin impairs vascular smooth muscle cell growth via the DNA damage response pathway
Source: Oncotarget. 2017 Mar 7;8(21):34045–56. doi: 10.18632/oncotarget.15973 (PMC5470950; doi:10.18632/oncotarget.15973)
Supplement: Supplementary file 4 [file oncotarget-08-34045-s004.docx]

| sample | W1,W2,W3,W4 | P1,P2,P3,P4 | |  |  |  |
| --- | --- | --- | --- | --- | --- | --- |
| MeasurementID | W (Processed Signal) | P (Processed Signal) | Fold Intensity | regulation | Q-Value | GENE_NAME |
| A_23_P10121 | 1.1222653 | -1.0933652 | 4.644845369 | down | 6.31E-05 | secreted frizzled-related protein 1 |
| A_23_P101407 | -2.5984368 | 2.2750416 | 29.313196 | up | 5.05E-04 | complement component 3 |
| A_23_P102364 | 1.1263759 | -1.0351338 | 4.47382795 | down | 2.83E-04 | neuronal guanine nucleotide exchange factor |
| A_23_P102611 | 1.4622462 | -1.2184049 | 6.411452393 | down | 0.001316656 | WNT1 inducible signaling pathway protein 2 |
| A_23_P103601 | 1.416776 | -1.5885901 | 8.029811414 | down | 3.69E-04 | mannosidase, alpha, class 1C, member 1 |
| A_23_P103877 | 1.107465 | -0.92779744 | 4.09897315 | down | 0.00166683 | leucine rich repeat containing 38 |
| A_23_P104073 | 0.8017895 | -0.81022644 | 3.056786846 | down | 7.88E-04 | S100 calcium binding protein A3 |
| A_23_P10542 | 1.6460173 | -1.7330652 | 10.40411562 | down | 4.46E-05 | HtrA serine peptidase 3 |
| A_23_P10647 | 1.1773746 | -1.083786 | 4.793769633 | down | 5.00E-04 | cytokine-like 1 |
| A_23_P108501 | 1.1589456 | -1.1195382 | 4.851678135 | down | 5.00E-04 | EPH receptor A4 |
| A_23_P108823 | -1.1403008 | 0.99527407 | 4.3941216 | up | 7.04E-04 | oxysterol binding protein-like 6 |
| A_23_P109269 | 0.94100964 | -0.89435637 | 3.568619303 | down | 1.93E-04 | laminin, alpha 5 |
| A_23_P111860 | 0.8948326 | -0.85522187 | 3.363712591 | down | 6.31E-05 | Ras association and DIL domains |
| A_23_P113613 | -1.4585474 | 1.2555287 | 6.561729 | up | 0.001832067 | CUB domain containing protein 1 |
| A_23_P114414 | -1.0810351 | 0.93490434 | 4.044439 | up | 0.001556291 | LON peptidase N-terminal domain and ring finger 3 |
| A_23_P114423 | 1.2688016 | -1.28306 | 5.863904064 | down | 7.46E-05 | regucalcin (senescence marker protein-30) |
| A_23_P114689 | 0.96512604 | -1.0256959 | 3.974633887 | down | 5.64E-04 | ArfGAP with SH3 domain, ankyrin repeat and PH domain 3 |
| A_23_P114883 | 1.4918189 | -1.1997037 | 6.45994832 | down | 0.001923565 | fibromodulin |
| A_23_P115064 | 1.2283618 | -1.1457777 | 5.184265381 | down | 2.42E-04 | cellular retinoic acid binding protein 2 |
| A_23_P116264 | 1.1065145 | -0.7707913 | 3.673883337 | down | 0.002302228 | neurogranin (protein kinase C substrate, RC3) |
| A_23_P120594 | 0.9412221 | -1.06149 | 4.007526937 | down | 5.06E-04 | acyl-CoA synthetase short-chain family member 1 |
| A_23_P121637 | -0.9093934 | 0.99659514 | 3.747656 | up | 2.22E-04 | protease, serine, 12 (neurotrypsin, motopsin) |
| A_23_P121885 | 1.2874718 | -0.91122234 | 4.590636635 | down | 0.003011333 | ropporin 1-like |
| A_23_P12363 | 0.9687958 | -0.8675462 | 3.571034482 | down | 0.001411297 | receptor tyrosine kinase-like orphan receptor 1 |
| A_23_P125423 | 1.2622502 | -0.9752302 | 4.715727683 | down | 0.003603906 | complement component 1, r subcomponent |
| A_23_P126089 | -1.1215664 | 1.1159703 | 4.7159114 | up | 2.53E-04 | small proline-rich protein 2C (pseudogene) |
| A_23_P12733 | 1.6842468 | -1.6878085 | 10.35356171 | down | 1.67E-04 | H2A histone family, member Y2 |
| A_23_P128174 | -1.0456678 | 0.99252915 | 4.107319 | up | 3.84E-04 | RAB3A interacting protein (rabin3) |
| A_23_P129005 | 1.0715485 | -1.1483887 | 4.658731821 | down | 0.002583588 | NYN domain and retroviral integrase containing |
| A_23_P13094 | -1.7889042 | 1.7835184 | 11.89615 | up | 1.02E-04 | matrix metallopeptidase 10 (stromelysin 2) |
| A_23_P131263 | -0.91880906 | 0.84545445 | 3.3970053 | up | 3.07E-04 | membrane protein, palmitoylated 4 (MAGUK p55 subfamily member 4) |
| A_23_P131676 | 1.7736065 | -1.4556234 | 9.377673223 | down | 0.001064865 | chemokine (C-X-C motif) receptor 7 |
| A_23_P132159 | 1.0115061 | -0.92203903 | 3.819927108 | down | 0.001979859 | ubiquitin specific peptidase 18 |
| A_23_P133293 | -1.8610101 | 1.4065697 | 9.630294 | up | 0.002755977 | multiple C2 domains, transmembrane 1 |
| A_23_P133408 | -4.1047945 | 3.312037 | 170.87903 | up | 0.004616794 | colony stimulating factor 2 (granulocyte-macrophage) |
| A_23_P134176 | -1.2594943 | 1.1443918 | 5.292268 | up | 9.22E-05 | superoxide dismutase 2, mitochondrial |
| A_23_P134835 | 0.89285135 | -0.903301 | 3.472927419 | down | 1.86E-04 | chondroitin sulfate N-acetylgalactosaminyltransferase 1 |
| A_23_P137016 | -0.8823416 | 0.84933496 | 3.3211355 | up | 9.99E-05 | spermidine/spermine N1-acetyltransferase 1 |
| A_23_P137470 | 0.9667978 | -0.9006635 | 3.648899397 | down | 9.57E-04 | signal-induced proliferation-associated 1 like 2 |
| A_23_P138680 | 0.9914439 | -0.8156514 | 3.499370288 | down | 0.001832067 | interleukin 15 receptor, alpha |
| A_23_P139143 | -0.85164106 | 0.80381036 | 3.1502175 | up | 3.05E-04 | syntaxin 3 |
| A_23_P139912 | 0.8914964 | -0.9561615 | 3.599154141 | down | 3.34E-04 | insulin-like growth factor binding protein 6 |
| A_23_P140427 | 0.98663807 | -0.9425149 | 3.808315304 | down | 1.83E-04 | Enah/Vasp-like |
| A_23_P140748 | 1.0301695 | -0.91495657 | 3.850714425 | down | 2.21E-04 | NDRG family member 4 |
| A_23_P141429 | 1.5105855 | -1.5034189 | 8.078035112 | down | 5.00E-04 | ABI family, member 3 |
| A_23_P142849 | -0.8220825 | 0.85045385 | 3.1877453 | up | 0.001626572 | Rho family GTPase 3 |
| A_23_P142872 | 1.773039 | -1.801672 | 11.91503153 | down | 9.10E-05 | transcription factor 7-like 1 (T-cell specific, HMG-box) |
| A_23_P14302 | 0.925858 | -0.94324684 | 3.653058779 | down | 5.53E-05 | chromosome 14 open reading frame 139 |
| A_23_P143190 | 0.95340943 | -0.8824322 | 3.5697959 | down | 3.21E-04 | v-myb myeloblastosis viral oncogene homolog (avian)-like 2 |
| A_23_P144476 | 1.1516762 | -1.0060843 | 4.462216959 | down | 0.001505462 | sprouty homolog 1, antagonist of FGF signaling (Drosophila) |
| A_23_P145114 | -1.1300719 | 0.850204 | 3.9456851 | up | 0.001593238 | glutamate-cysteine ligase, catalytic subunit |
| A_23_P14515 | 0.85849285 | -0.9162197 | 3.421698234 | down | 3.34E-04 | acyl-CoA thioesterase 4 |
| A_23_P145935 | 0.98292935 | -1.2605149 | 4.735261699 | down | 0.004179766 | EPH receptor B6 |
| A_23_P146922 | -1.1027968 | 1.163152 | 4.809706 | up | 2.57E-04 | growth arrest-specific 6 |
| A_23_P149121 | -1.1395619 | 1.1268532 | 4.811261 | up | 2.50E-04 | DIRAS family, GTP-binding RAS-like 3 |
| A_23_P149281 | -1.0713451 | 1.1139034 | 4.548051 | up | 8.68E-05 | EPH receptor A2 |
| A_23_P149545 | -1.0059456 | 0.890259 | 3.7223265 | up | 0.004631132 | histone cluster 2, H2be |
| A_23_P150281 | 1.1101408 | -1.2391254 | 5.095649676 | down | 3.63E-04 | tumor protein p53 inducible protein 11 |
| A_23_P150609 | 0.834522 | -0.7991755 | 3.103072911 | down | 0.001919003 | insulin-like growth factor 2 (somatomedin A) |
| A_23_P15146 | -0.7760948 | 0.8754078 | 3.1416068 | up | 0.001919003 | interleukin 32 |
| A_23_P151710 | 1.4644558 | -1.4021052 | 7.293245426 | down | 2.85E-05 | prostaglandin E receptor 2 (subtype EP2), 53kDa |
| A_23_P151975 | -1.0377737 | 1.0008729 | 4.1085997 | up | 0.001150909 | Rh family, C glycoprotein |
| A_23_P152002 | -2.2712026 | 2.1308076 | 21.14156 | up | 5.20E-05 | BCL2-related protein A1 |
| A_23_P152791 | -1.5992883 | 0.7154459 | 4.97513 | up | 0.023543756 | solute carrier family 16, member 6 (monocarboxylic acid transporter 7) |
| A_23_P154605 | 2.0327106 | -2.417928 | 21.86632399 | down | 4.15E-04 | sulfatase 2 |
| A_23_P155755 | -1.1788418 | 1.0345032 | 4.6374927 | up | 0.002980595 | chemokine (C-X-C motif) ligand 6 (granulocyte chemotactic protein 2) |
| A_23_P156708 | 1.1467674 | -0.95624757 | 4.296062457 | down | 0.002117146 | tenascin XB |
| A_23_P159952 | -1.8255439 | 1.689168 | 11.42967 | up | 9.97E-05 | brain expressed, X-linked 1 |
| A_23_P161218 | -1.6331618 | 1.4998959 | 8.7729225 | up | 1.32E-04 | ankyrin repeat domain 1 (cardiac muscle) |
| A_23_P161698 | -5.4848156 | 4.940816 | 1375.3961 | up | 8.17E-04 | matrix metallopeptidase 3 (stromelysin 1, progelatinase) |
| A_23_P161727 | 1.0202134 | -0.86956334 | 3.705778665 | down | 0.001926898 | heat shock 27kDa protein 2 |
| A_23_P16275 | 1.4575638 | -1.569313 | 8.15043446 | down | 0.001444966 | testis-specific serine kinase substrate |
| A_23_P163402 | -0.9063997 | 0.8797324 | 3.44889 | up | 3.29E-04 | cytochrome P450, family 1, subfamily A, polypeptide 1 |
| A_23_P164057 | 1.3850389 | -1.0789301 | 5.51732509 | down | 0.003097973 | microfibrillar-associated protein 4 |
| A_23_P165783 | -1.0591307 | 0.9589534 | 4.050455 | up | 0.002547233 | melanophilin |
| A_23_P166087 | 1.4972677 | -1.3983917 | 7.441840528 | down | 5.69E-05 | Ras association (RalGDS/AF-6) domain family member 2 |
| A_23_P166297 | 0.68165624 | -1.6362059 | 4.985927966 | down | 0.024313081 | ATP-binding cassette, sub-family G (WHITE), member 1 |
| A_23_P166797 | 1.4290576 | -1.3851358 | 7.033258875 | down | 1.71E-04 | receptor (chemosensory) transporter protein 4 |
| A_23_P166848 | -0.9785664 | 0.86820674 | 3.5969477 | up | 0.001059866 | lactotransferrin |
| A_23_P167367 | -1.5387561 | 1.3720733 | 7.520505 | up | 8.27E-04 | paired-like homeodomain 2 |
| A_23_P167983 | -1.0362378 | 0.9798002 | 4.044715 | up | 5.89E-04 | histone cluster 1, H2ac |
| A_23_P168610 | -1.4376636 | 1.3997506 | 7.147379 | up | 1.07E-04 | tetraspanin 13 |
| A_23_P17663 | 1.930764 | -1.7391291 | 12.72764021 | down | 4.35E-04 | myxovirus (influenza virus) resistance 1, interferon-inducible protein p78 (mouse) |
| A_23_P1782 | -1.4313935 | 1.5160203 | 7.713651 | up | 6.02E-05 | CD82 molecule |
| A_23_P18017 | -1.447836 | 1.3253056 | 6.835949 | up | 0.002011299 | carboxypeptidase A3 (mast cell) |
| A_23_P1962 | 1.4771059 | -1.3145657 | 6.924315843 | down | 0.001047941 | retinoic acid receptor responder (tazarotene induced) 3 |
| A_23_P200710 | 0.8162048 | -0.7978232 | 3.061052883 | down | 9.11E-05 | phosphoinositide-3-kinase, class 2, beta polypeptide |
| A_23_P201459 | 2.0124943 | -1.8311765 | 14.35688547 | down | 2.53E-04 | interferon, alpha-inducible protein 6 |
| A_23_P202071 | -0.9656141 | 0.70788217 | 3.189867 | up | 0.004791769 | CUG triplet repeat, RNA binding protein 2 |
| A_23_P202448 | 1.6802723 | -1.5744479 | 9.544834522 | down | 4.37E-05 | chemokine (C-X-C motif) ligand 12 (stromal cell-derived factor 1) |
| A_23_P204087 | 1.3705155 | -1.6505351 | 8.117584181 | down | 6.45E-04 | 2'-5'-oligoadenylate synthetase 2, 69/71kDa |
| A_23_P204751 | 0.8978975 | -1.0543857 | 3.869864656 | down | 0.004718687 | amiloride-sensitive cation channel 2, neuronal |
| A_23_P205370 | -0.9127561 | 0.9004718 | 3.514277 | up | 0.002804825 | ankyrin repeat and SOCS box-containing 2 |
| A_23_P206733 | -1.2030247 | 0.9484198 | 4.4427238 | up | 0.003306507 | carboxylesterase 1 (monocyte/macrophage serine esterase 1) |
| A_23_P207507 | -1.9279718 | 2.0619094 | 15.888172 | up | 4.45E-05 | ATP-binding cassette, sub-family C (CFTR/MRP), member 3 |
| A_23_P208210 | -0.9394139 | 0.8174387 | 3.3796003 | up | 0.001235385 | zinc finger protein 432 |
| A_23_P210465 | -1.1622517 | 1.5553256 | 6.5776725 | up | 0.001981361 | peptidase inhibitor 3, skin-derived |
| A_23_P211522 | 1.0851877 | -1.0894849 | 4.514832897 | down | 2.55E-04 | synaptogyrin 1 |
| A_23_P211631 | 1.0238733 | -1.0527298 | 4.218128716 | down | 3.02E-05 | fibulin 1 |
| A_23_P21207 | 0.7941239 | -0.82739496 | 3.07698812 | down | 6.72E-04 | ubiquitin-like modifier activating enzyme 7 |
| A_23_P212089 | -1.3207706 | 1.207886 | 5.7703404 | up | 0.001305438 | nuclear factor of kappa light polypeptide gene enhancer in B-cells inhibitor, zeta |
| A_23_P21363 | 0.8796761 | -0.8217652 | 3.252256741 | down | 5.05E-04 | AHNAK nucleoprotein |
| A_23_P214026 | 1.050461 | -0.973475 | 4.066918376 | down | 0.005371906 | fibrillin 2 |
| A_23_P214079 | -0.88434327 | 0.7883327 | 3.1880538 | up | 0.001003974 | serine peptidase inhibitor, Kazal type 1 |
| A_23_P214144 | -2.8255162 | 3.115306 | 61.42788 | up | 6.31E-05 | collagen, type X, alpha 1 |
| A_23_P214821 | -1.7948703 | 1.6366756 | 10.789422 | up | 0.001269367 | endothelin 1 |
| A_23_P215634 | 1.7004538 | -1.1982565 | 7.457593699 | down | 0.007068482 | insulin-like growth factor binding protein 3 |
| A_23_P215900 | 0.88787556 | -0.82847 | 3.286029838 | down | 2.22E-04 | scavenger receptor class A, member 3 |
| A_23_P216023 | -1.038451 | 0.7414777 | 3.434092 | up | 0.003642844 | angiopoietin 1 |
| A_23_P216966 | 0.7646539 | -0.86867356 | 3.102276792 | down | 0.002319397 | prostaglandin-endoperoxide synthase 1 (prostaglandin G/H synthase and cyclooxygenase) |
| A_23_P217319 | 2.5248854 | -2.701157 | 37.42790216 | down | 4.38E-04 | fibroblast growth factor 13 |
| A_23_P22134 | -1.937252 | 1.8715496 | 14.014046 | up | 3.02E-05 | basonuclin 1 |
| A_23_P22350 | -0.84947765 | 0.8326297 | 3.2089634 | up | 3.77E-04 | GRAM domain containing 3 |
| A_23_P2283 | -0.9188857 | 0.83082986 | 3.3629227 | up | 3.62E-04 | tachykinin 3 |
| A_23_P24077 | 0.9695921 | -1.0122511 | 3.949974365 | down | 0.002695 | chromosome 10 open reading frame 54 |
| A_23_P24716 | -1.0129423 | 1.1950593 | 4.6203485 | up | 3.34E-04 | transmembrane protein 132A |
| A_23_P250358 | 1.0386679 | -0.99373376 | 4.090852767 | down | 6.32E-05 | hect domain and RLD 6 |
| A_23_P251499 | 0.9307976 | -0.84565973 | 3.425838979 | down | 4.65E-04 | procollagen C-endopeptidase enhancer |
| A_23_P252062 | 1.1961248 | -1.227617 | 5.365608542 | down | 4.82E-05 | peroxisome proliferator-activated receptor gamma |
| A_23_P252541 | 1.1374819 | -1.1053995 | 4.733414565 | down | 4.82E-05 | RAB7B, member RAS oncogene family |
| A_23_P253221 | -0.9405632 | 0.9994087 | 3.8369818 | up | 4.16E-04 | Rho guanine nucleotide exchange factor (GEF) 4 |
| A_23_P254741 | 1.1136844 | -0.88026714 | 3.983265188 | down | 8.88E-04 | superoxide dismutase 3, extracellular |
| A_23_P257003 | 1.1758821 | -1.079702 | 4.775276446 | down | 0.001145916 | proprotein convertase subtilisin/kexin type 5 |
| A_23_P257649 | 1.5715585 | -1.836299 | 10.61371245 | down | 7.71E-04 | retinol binding protein 1, cellular |
| A_23_P258769 | 1.5110216 | -1.2603073 | 6.827365556 | down | 0.001353468 | major histocompatibility complex, class II, DP beta 1 |
| A_23_P259071 | -1.1417129 | 1.1750429 | 4.982106 | up | 0.00179086 | amphiregulin |
| A_23_P259442 | 1.1530533 | -0.8257549 | 3.941673325 | down | 0.005632348 | carboxypeptidase E |
| A_23_P26024 | -2.2154083 | 2.068947 | 19.485853 | up | 9.27E-04 | chromosome 15 open reading frame 48 |
| A_23_P26124 | -1.3120487 | 1.3167602 | 6.1851516 | up | 5.33E-04 | RAR-related orphan receptor A |
| A_23_P27315 | 1.072628 | -1.1140356 | 4.552514668 | down | 2.85E-05 | elastin microfibril interfacer 2 |
| A_23_P29124 | 1.1387248 | -1.1167543 | 4.774928493 | down | 4.46E-05 | septin 5 |
| A_23_P29939 | -2.1406221 | 2.1432142 | 19.478848 | up | 2.85E-05 | synuclein, alpha (non A4 component of amyloid precursor) |
| A_23_P301247 | 1.1910264 | -0.7458403 | 3.828732378 | down | 0.015449701 | histone cluster 2, H2ac |
| A_23_P302470 | 1.356322 | -1.4720839 | 7.102888176 | down | 9.22E-05 | sulfotransferase family, cytosolic, 1B, member 1 |
| A_23_P30567 | 1.1980104 | -1.4441376 | 6.242604465 | down | 0.001899277 | corticotropin releasing hormone binding protein |
| A_23_P30655 | -0.8443923 | 0.7617831 | 3.044437 | up | 0.001389468 | nuclear factor of kappa light polypeptide gene enhancer in B-cells inhibitor, epsilon |
| A_23_P315364 | -2.0396333 | 1.9085863 | 15.43592 | up | 2.23E-04 | chemokine (C-X-C motif) ligand 2 |
| A_23_P316612 | 1.0334034 | -1.0871468 | 4.34859749 | down | 1.09E-04 | GLIS family zinc finger 1 |
| A_23_P321846 | -0.77942336 | 0.9258604 | 3.2609305 | up | 0.001835416 | potassium voltage-gated channel, delayed-rectifier, subfamily S, member 1 |
| A_23_P32414 | -1.071758 | 0.95900965 | 4.086222 | up | 4.38E-04 | serine/threonine protein kinase MST4 |
| A_23_P3312 | 0.8376858 | -0.8402877 | 3.199781903 | down | 3.02E-04 | immunoglobulin superfamily containing leucine-rich repeat |
| A_23_P334870 | -0.94490147 | 0.82716334 | 3.4154243 | up | 5.01E-04 | transmembrane protein 217 |
| A_23_P337729 | 0.8990567 | -0.86992323 | 3.40812881 | down | 3.20E-04 | transmembrane protein 180 |
| A_23_P339098 | -1.1599997 | 1.0751288 | 4.708046 | up | 8.60E-04 | solute carrier family 35, member F2 |
| A_23_P342138 | 0.84300256 | -0.97584176 | 3.527984591 | down | 5.77E-04 | ADAMTS-like 1 |
| A_23_P34375 | 1.0100815 | -1.0661007 | 4.216898716 | down | 4.45E-05 | transcription elongation factor A (SII), 3 |
| A_23_P348257 | -0.9819057 | 0.91572714 | 3.7260134 | up | 1.96E-04 | NUAK family, SNF1-like kinase, 1 |
| A_23_P355517 | -1.8077773 | 1.6772091 | 11.196581 | up | 2.79E-04 | synaptopodin 2-like |
| A_23_P362694 | -2.3017256 | 1.9096949 | 18.525242 | up | 8.78E-04 | chromosome 4 open reading frame 7 |
| A_23_P363174 | 0.9877479 | -0.75181293 | 3.339334897 | down | 0.011114043 | histone cluster 1, H2al |
| A_23_P366366 | 0.88199925 | -0.81414247 | 3.240332339 | down | 5.28E-05 | secernin 1 |
| A_23_P368484 | 0.9793855 | -1.0511031 | 4.085431604 | down | 1.94E-04 | chromosome 17 open reading frame 76 |
| A_23_P369994 | 1.0644228 | -0.9399028 | 4.01201132 | down | 0.002616131 | doublecortin-like kinase 1 |
| A_23_P373031 | 0.8578911 | -0.8941355 | 3.368313834 | down | 0.002963833 | calcium channel, voltage-dependent, L type, alpha 1C subunit |
| A_23_P374844 | 1.2530165 | -1.0792607 | 5.035996548 | down | 5.80E-04 | galanin prepropeptide |
| A_23_P383819 | -0.93202364 | 0.66661644 | 3.028577 | up | 0.006193321 | T-box 3 |
| A_23_P38567 | -2.2602687 | 2.1454592 | 21.196114 | up | 3.19E-04 | cytospin B |
| A_23_P38732 | -1.2594692 | 1.2357633 | 5.6381917 | up | 2.35E-05 | cadherin 2, type 1, N-cadherin (neuronal) |
| A_23_P388900 | -1.2865087 | 0.8559623 | 4.4151754 | up | 0.003130996 | solute carrier family 22, member 15 |
| A_23_P388993 | -0.9881495 | 0.751024 | 3.3384387 | up | 0.001983161 | zinc finger CCCH-type containing 12C |
| A_23_P389987 | 0.87961364 | -0.73905396 | 3.07091297 | down | 0.001732432 | T-cell leukemia homeobox 2 |
| A_23_P393034 | -1.2131451 | 1.1823374 | 5.2615304 | up | 3.20E-04 | hyaluronan synthase 3 |
| A_23_P39465 | 1.823978 | -1.7180061 | 11.64778813 | down | 6.94E-04 | bone marrow stromal cell antigen 2 |
| A_23_P3956 | -0.85543966 | 0.9065008 | 3.39154 | up | 1.05E-04 | C1q and tumor necrosis factor related protein 1 |
| A_23_P39955 | -2.625468 | 2.4143906 | 32.896423 | up | 0.001113062 | actin, gamma 2, smooth muscle, enteric |
| A_23_P401774 | -1.6630749 | 0.9844191 | 6.265779 | up | 0.010855983 | ELMO/CED-12 domain containing 1 |
| A_23_P41114 | -0.967921 | 0.87390924 | 3.584645 | up | 0.001919003 | cystatin A (stefin A) |
| A_23_P41344 | -3.0864067 | 2.0377772 | 34.87652 | up | 0.005224033 | epiregulin |
| A_23_P41476 | 1.0533943 | -1.0241063 | 4.220753691 | down | 5.53E-05 | shisa homolog 3 (Xenopus laevis) |
| A_23_P415021 | 1.2315133 | -1.2731386 | 5.675124437 | down | 6.31E-05 | methyltransferase like 7A |
| A_23_P415652 | 0.80746984 | -0.78413653 | 3.013847423 | down | 1.22E-04 | UDP-N-acetyl-alpha-D-galactosamine:polypeptide N-acetylgalactosaminyltransferase 12 (GalNAc-T12) |
| A_23_P416894 | -1.0530393 | 1.2711788 | 5.0079436 | up | 0.003539664 | pigeon homolog (Drosophila) |
| A_23_P417918 | 1.3601499 | -1.5272528 | 7.399370595 | down | 6.77E-04 | proenkephalin |
| A_23_P41804 | 1.2776825 | -1.3033373 | 5.983625092 | down | 7.46E-05 | naked cuticle homolog 2 (Drosophila) |
| A_23_P421011 | 1.17803 | -1.1392746 | 4.984002101 | down | 7.29E-04 | Kazal-type serine peptidase inhibitor domain 1 |
| A_23_P422212 | -1.2407486 | 1.2934849 | 5.7926903 | up | 1.96E-04 | solute carrier family 35, member F3 |
| A_23_P42257 | -1.4777968 | 1.3710947 | 7.204466 | up | 0.002734854 | immediate early response 3 |
| A_23_P425681 | -1.5519348 | 1.5173705 | 8.393691 | up | 1.12E-04 | cholecystokinin |
| A_23_P42868 | 0.94902515 | -1.0428874 | 3.977639303 | down | 0.001132522 | insulin-like growth factor binding protein 1 |
| A_23_P43164 | 1.2424128 | -1.1730003 | 5.334722353 | down | 0.003043562 | sulfatase 1 |
| A_23_P43276 | 1.0124652 | -1.0362742 | 4.137442873 | down | 4.64E-04 | G protein-coupled receptor 124 |
| A_23_P433016 | 1.1117802 | -1.0387778 | 4.439994583 | down | 1.83E-04 | fibulin 1 |
| A_23_P44674 | 1.4347336 | -1.5322056 | 7.818756227 | down | 1.28E-04 | cysteine-rich protein 1 (intestinal) |
| A_23_P4551 | 1.2569113 | -1.1663895 | 5.363968315 | down | 4.93E-04 | SET binding protein 1 |
| A_23_P45871 | 1.4550054 | -1.3083718 | 6.789837948 | down | 0.001368163 | interferon-induced protein 44-like |
| A_23_P46936 | -0.9942261 | 1.088109 | 4.234921 | up | 4.43E-04 | early growth response 2 |
| A_23_P47924 | -1.703411 | 1.2613236 | 7.8068175 | up | 0.002354975 | protein tyrosine phosphatase, receptor type, R |
| A_23_P4808 | 0.98986673 | -0.96008015 | 3.863603216 | down | 6.92E-04 | prostaglandin E receptor 1 (subtype EP1), 42kDa |
| A_23_P49009 | 1.045299 | -1.0288414 | 4.210934821 | down | 1.90E-04 | lysophosphatidylcholine acyltransferase 4 |
| A_23_P500381 | -1.0439136 | 0.879539 | 3.7932978 | up | 3.36E-04 | 5-hydroxytryptamine (serotonin) receptor 7 (adenylate cyclase-coupled) |
| A_23_P501007 | 0.99758816 | -0.8597305 | 3.623335965 | down | 0.002690629 | EGF-containing fibulin-like extracellular matrix protein 1 |
| A_23_P501634 | -0.90033305 | 0.85985994 | 3.3874342 | up | 7.99E-04 | butyrophilin, subfamily 2, member A1 |
| A_23_P502343 | 1.221049 | -0.8864955 | 4.30957152 | down | 0.014902537 | ADAM metallopeptidase domain 33 |
| A_23_P50946 | 0.8829229 | -0.8177191 | 3.250455706 | down | 0.001998112 | receptor (G protein-coupled) activity modifying protein 1 |
| A_23_P51126 | 0.8796518 | -0.8088454 | 3.223207922 | down | 8.53E-04 | interleukin 1 receptor-like 1 |
| A_23_P51187 | 1.0137019 | -1.2386663 | 4.764643297 | down | 6.96E-04 | protein kinase C, zeta |
| A_23_P51376 | -1.3574828 | 1.167428 | 5.755379 | up | 5.89E-04 | Na+/K+ transporting ATPase interacting 1 |
| A_23_P52986 | -0.83160615 | 0.82172894 | 3.1455996 | up | 7.63E-04 | von Willebrand factor C and EGF domains |
| A_23_P53137 | 2.386888 | -2.2738447 | 25.29416673 | down | 5.64E-04 | hemoglobin, gamma A |
| A_23_P53588 | -1.0585907 | 1.0945776 | 4.4480352 | up | 1.97E-04 | wingless-type MMTV integration site family, member 5B |
| A_23_P53663 | -1.246107 | 0.52273345 | 3.4077995 | up | 0.027382873 | PRKC, apoptosis, WT1, regulator |
| A_23_P55749 | 1.2164848 | -1.2532077 | 5.539257214 | down | 6.72E-04 | collagen, type V, alpha 3 |
| A_23_P56559 | 2.8495817 | -2.57646 | 42.99335194 | down | 1.38E-04 | dehydrogenase/reductase (SDR family) member 9 |
| A_23_P57199 | -1.0315521 | 0.8474064 | 3.6780944 | up | 0.004046933 | gamma-glutamyltransferase light chain 1 |
| A_23_P57709 | 1.1109436 | -1.1049683 | 4.645751031 | down | 7.46E-05 | procollagen C-endopeptidase enhancer 2 |
| A_23_P58251 | 1.386018 | -1.363029 | 6.722729299 | down | 1.55E-04 | carboxypeptidase Z |
| A_23_P59807 | 1.7472277 | -1.8271127 | 11.91197205 | down | 3.34E-04 | wingless-type MMTV integration site family member 2 |
| A_23_P62081 | -1.4485172 | 1.487256 | 7.6516633 | up | 1.18E-04 | secretogranin V (7B2 protein) |
| A_23_P62188 | 0.86866164 | -0.78353 | 3.143107442 | down | 2.11E-04 | zinc finger, C4H2 domain containing |
| A_23_P6263 | 2.2871954 | -2.2712026 | 23.56213264 | down | 1.77E-04 | myxovirus (influenza virus) resistance 2 (mouse) |
| A_23_P62752 | -2.0532436 | 1.8882236 | 15.363844 | up | 1.22E-04 | natriuretic peptide precursor B |
| A_23_P62901 | 0.88243103 | -0.9398811 | 3.536475062 | down | 1.38E-04 | BTG family, member 2 |
| A_23_P63660 | 1.2916942 | -0.9987296 | 4.891998374 | down | 0.001150528 | chromosome 10 open reading frame 58 |
| A_23_P64828 | 2.054779 | -1.9032532 | 15.54126657 | down | 3.67E-04 | 2',5'-oligoadenylate synthetase 1, 40/46kDa |
| A_23_P64873 | 1.4303145 | -1.0806205 | 5.699893971 | down | 0.002690629 | decorin |
| A_23_P65442 | 0.96018195 | -0.81788754 | 3.429669315 | down | 7.66E-04 | interferon regulatory factor 9 |
| A_23_P66798 | -1.6853895 | 1.6420312 | 10.038144 | up | 5.01E-04 | keratin 19 |
| A_23_P67661 | 1.2112532 | -1.2151289 | 5.375436909 | down | 5.83E-04 | cytochrome c oxidase subunit VIIa polypeptide 1 (muscle) |
| A_23_P67847 | 1.830925 | -1.7218807 | 11.7354859 | down | 5.53E-05 | UDP-N-acetyl-alpha-D-galactosamine:polypeptide N-acetylgalactosaminyltransferase 14 (GalNAc-T14) |
| A_23_P68031 | -1.5776716 | 1.5518287 | 8.751319 | up | 5.42E-05 | signal transducer and activator of transcription 4 |
| A_23_P69326 | 2.3036098 | -2.3641539 | 25.41773734 | down | 7.93E-06 | Ca++-dependent secretion activator |
| A_23_P69383 | 1.1249332 | -0.88381386 | 4.024325761 | down | 0.00149673 | poly (ADP-ribose) polymerase family, member 9 |
| A_23_P69497 | 2.2170186 | -2.1609826 | 20.792643 | down | 3.02E-05 | C-type lectin domain family 3, member B |
| A_23_P70818 | 1.6281693 | -1.5975777 | 9.355061149 | down | 3.02E-05 | smoothened homolog (Drosophila) |
| A_23_P71037 | -1.2241836 | 1.2910621 | 5.71695 | up | 1.74E-04 | interleukin 6 (interferon, beta 2) |
| A_23_P7144 | -1.0932806 | 1.0953925 | 4.55886 | up | 0.003411895 | chemokine (C-X-C motif) ligand 1 (melanoma growth stimulating activity, alpha) |
| A_23_P71530 | 1.3002121 | -1.2490153 | 5.853207754 | down | 1.90E-04 | tumor necrosis factor receptor superfamily, member 11b |
| A_23_P72668 | 0.79405737 | -0.85818875 | 3.143226391 | down | 9.96E-04 | serum deprivation response |
| A_23_P72737 | 2.2569797 | -2.2070367 | 22.07002634 | down | 7.20E-05 | interferon induced transmembrane protein 1 (9-27) |
| A_23_P73420 | 0.8420539 | -0.8297198 | 3.18606045 | down | 0.001561416 | trafficking protein particle complex 9 |
| A_23_P73429 | -1.2045768 | 1.2733266 | 5.570873 | up | 1.12E-04 | hematopoietic cell-specific Lyn substrate 1 |
| A_23_P74290 | -1.4394702 | 1.2385834 | 6.3999176 | up | 0.001003974 | guanylate binding protein 5 |
| A_23_P75283 | 1.3473656 | -1.3201392 | 6.353293837 | down | 1.96E-04 | retinol binding protein 4, plasma |
| A_23_P75786 | 0.9025929 | -0.91350174 | 3.521267115 | down | 7.82E-04 | solute carrier family 15, member 3 |
| A_23_P76749 | 0.9624753 | -1.0480354 | 4.029248151 | down | 0.001090833 | UDP-N-acetyl-alpha-D-galactosamine:polypeptide N-acetylgalactosaminyltransferase-like 1 |
| A_23_P76901 | 1.1658211 | -1.1110679 | 4.846317223 | down | 8.30E-05 | pleckstrin homology domain containing, family G (with RhoGef domain) member 3 |
| A_23_P78742 | 1.2522988 | -1.1276084 | 5.205032475 | down | 3.28E-04 | fms-related tyrosine kinase 3 ligand |
| A_23_P78762 | -0.9193839 | 0.8739157 | 3.466067 | up | 9.96E-04 | hydroxysteroid (17-beta) dehydrogenase 14 |
| A_23_P79518 | -1.4243879 | 1.2967374 | 6.5938697 | up | 0.003346009 | interleukin 1, beta |
| A_23_P82929 | 1.6591554 | -1.5138578 | 9.01928612 | down | 1.77E-04 | nephroblastoma overexpressed gene |
| A_23_P83298 | 0.83969903 | -0.7731674 | 3.058589421 | down | 0.001547269 | paired related homeobox 2 |
| A_23_P87150 | -1.3022864 | 1.214138 | 5.721623 | up | 0.002899002 | leupaxin |
| A_23_P87545 | 1.0810084 | -1.0405095 | 4.351515226 | down | 5.44E-04 | interferon induced transmembrane protein 3 (1-8U) |
| A_23_P89431 | -0.91579175 | 0.83368516 | 3.3623662 | up | 5.06E-04 | chemokine (C-C motif) ligand 2 |
| A_23_P8981 | 1.6536999 | -1.6179452 | 9.657468334 | down | 1.55E-04 | steroidogenic acute regulatory protein |
| A_23_P90722 | -0.82064795 | 0.94644916 | 3.4036841 | up | 3.07E-04 | protein tyrosine phosphatase, receptor type, N |
| A_23_P90925 | -2.239828 | 2.188521 | 21.531084 | up | 6.11E-05 | interleukin 1 family, member 8 (eta) |
| A_23_P91334 | 1.3123021 | -1.574176 | 7.394630034 | down | 0.001365112 | heat shock 70kD protein 12B |
| A_23_P91512 | -1.7834892 | 1.3993554 | 9.080959 | up | 0.00230271 | claudin 14 |
| A_23_P93938 | 1.1794562 | -1.0155683 | 4.578974512 | down | 5.89E-04 | NAC alpha domain containing |
| A_23_P94103 | 1.9100652 | -1.8627461 | 13.66876856 | down | 4.45E-05 | scavenger receptor class A, member 5 (putative) |
| A_23_P94338 | 0.97192955 | -0.8015032 | 3.418664335 | down | 0.005931197 | ectonucleotide pyrophosphatase/phosphodiesterase 2 |
| A_23_P94800 | 0.99587727 | -0.76052904 | 3.378554839 | down | 0.005401544 | S100 calcium binding protein A4 |
| A_23_P96369 | -0.9288833 | 1.1713454 | 4.2877736 | up | 5.89E-04 | chromosome X open reading frame 57 |
| A_23_P97606 | 0.8349731 | -0.83459926 | 3.181202706 | down | 8.78E-04 | glutathione S-transferase mu 5 |
| A_23_P98350 | -2.4960642 | 2.0149667 | 22.801094 | up | 8.39E-04 | baculoviral IAP repeat-containing 3 |
| A_23_P98645 | 0.81987214 | -0.81644416 | 3.108710358 | down | 0.003447495 | dachsous 1 (Drosophila) |
| A_23_P9883 | -0.9120821 | 0.83914995 | 3.3664594 | up | 0.003020714 | NLR family, pyrin domain containing 3 |
| A_24_P100517 | 0.76415944 | -0.85137653 | 3.064254036 | down | 4.80E-04 | chromosome 9 open reading frame 140 |
| A_24_P11315 | 0.94875073 | -0.73634243 | 3.215611718 | down | 0.002225534 | olfactomedin-like 3 |
| A_24_P11506 | -1.3085651 | 1.3861101 | 6.47408 | up | 4.65E-04 | kynureninase (L-kynurenine hydrolase) |
| A_24_P120537 | -1.1371882 | 1.1234938 | 4.79218 | up | 2.00E-04 | SH3 domain containing ring finger 2 |
| A_24_P12401 | -0.80308604 | 0.8281305 | 3.097741 | up | 5.05E-04 | vascular endothelial growth factor A |
| A_24_P142118 | -0.95989656 | 0.6585741 | 3.0704937 | up | 0.005505583 | thrombospondin 1 |
| A_24_P15502 | 1.1532171 | -1.0652313 | 4.653926357 | down | 5.92E-04 |  |
| A_24_P160401 | -1.3928798 | 1.249074 | 6.2417636 | up | 2.39E-04 | CUB domain containing protein 1 |
| A_24_P16124 | 1.1989768 | -1.1082096 | 4.949169309 | down | 3.34E-04 | interferon induced transmembrane protein 4 pseudogene |
| A_24_P16214 | -1.003369 | 0.7682276 | 3.414316 | up | 0.001062612 | hypothetical protein LOC100127980 |
| A_24_P166443 | 1.5694139 | -1.3490262 | 7.560282479 | down | 8.66E-04 | major histocompatibility complex, class II, DP beta 1 |
| A_24_P179816 | 0.81199956 | -0.90496063 | 3.287429854 | down | 0.001590924 | solute carrier family 27 (fatty acid transporter), member 3 |
| A_24_P183150 | -1.9117328 | 1.8802583 | 13.851701 | up | 2.23E-04 | chemokine (C-X-C motif) ligand 3 |
| A_24_P192727 | 0.98813725 | -0.9595561 | 3.857572558 | down | 5.01E-04 |  |
| A_24_P212481 | -1.3184328 | 1.2878864 | 6.089481 | up | 4.45E-05 | multiple C2 domains, transmembrane 1 |
| A_24_P215765 | 1.238466 | -1.2705084 | 5.692153321 | down | 4.45E-05 | ATPase, class V, type 10A |
| A_24_P228796 | 1.1007512 | -1.0409696 | 4.41288097 | down | 4.97E-04 | G antigen 7 |
| A_24_P237270 | -0.93598974 | 0.9512086 | 3.6991615 | up | 3.28E-04 | adenosine A2a receptor |
| A_24_P250922 | -1.3475283 | 1.3646593 | 6.5531464 | up | 3.18E-04 | prostaglandin-endoperoxide synthase 2 (prostaglandin G/H synthase and cyclooxygenase) |
| A_24_P252996 | -0.8448626 | 0.93552804 | 3.4351916 | up | 4.32E-04 | folate receptor 3 (gamma) |
| A_24_P257416 | -1.2477434 | 1.1996636 | 5.454349 | up | 3.66E-04 | chemokine (C-X-C motif) ligand 2 |
| A_24_P260639 | 1.2771852 | -0.8063855 | 4.238549643 | down | 0.013616687 | histone cluster 1, H1d |
| A_24_P262127 | -1.4946 | 1.2303004 | 6.6111465 | up | 0.001009482 | Ras-related associated with diabetes |
| A_24_P270460 | 1.6927004 | -1.579493 | 9.661140333 | down | 3.96E-04 | interferon, alpha-inducible protein 27 |
| A_24_P272761 | 0.86506975 | -0.8711101 | 3.331518211 | down | 0.001680561 | DENN/MADD domain containing 1A |
| A_24_P277367 | -2.9206693 | 3.0577464 | 63.049606 | up | 9.97E-05 | chemokine (C-X-C motif) ligand 5 |
| A_24_P27977 | 0.97150755 | -0.78174305 | 3.371172724 | down | 0.002650594 | transient receptor potential cation channel, subfamily M, member 2 |
| A_24_P287043 | 1.161905 | -1.0803449 | 4.731343661 | down | 3.96E-04 | interferon induced transmembrane protein 2 (1-8D) |
| A_24_P28722 | 1.4584496 | -1.5910667 | 8.279342362 | down | 3.20E-04 | radical S-adenosyl methionine domain containing 2 |
| A_24_P291826 | -1.0663613 | 0.92390513 | 3.9731038 | up | 0.002512733 | synaptotagmin-like 3 |
| A_24_P3005 | -0.95201814 | 0.70021296 | 3.1431935 | up | 0.006177067 | sodium channel, voltage-gated, type IX, alpha subunit |
| A_24_P300777 | -1.3903023 | 1.269352 | 6.3188157 | up | 2.21E-04 | ADAM metallopeptidase domain 8 |
| A_24_P304154 | -1.426415 | 1.3925831 | 7.0567217 | up | 5.67E-05 | adenosine monophosphate deaminase (isoform E) |
| A_24_P305784 | -1.7539726 | 1.6159499 | 10.338268 | up | 7.09E-04 | SPANX family, member B2 |
| A_24_P308029 | 0.96260905 | -1.1281884 | 4.259834574 | down | 0.00805029 | heat shock protein, alpha-crystallin-related, B6 |
| A_24_P317762 | 1.1026449 | -1.0196424 | 4.353836653 | down | 5.05E-04 | lymphocyte antigen 6 complex, locus E |
| A_24_P335305 | 1.1096716 | -1.1155807 | 4.67592606 | down | 4.82E-05 | 2'-5'-oligoadenylate synthetase 3, 100kDa |
| A_24_P34155 | -0.9478049 | 0.9686992 | 3.7750719 | up | 3.26E-04 | runt-related transcription factor 1 |
| A_24_P349196 | -1.0734823 | 1.0011361 | 4.21233 | up | 5.01E-04 | coiled-coil domain containing 30 |
| A_24_P353905 | 0.8535321 | -0.84129214 | 3.237374531 | down | 4.43E-04 | matrix-remodelling associated 8 |
| A_24_P366526 | -1.1012056 | 0.9823439 | 4.2384872 | up | 0.00347395 | synaptogyrin 2 |
| A_24_P378019 | 1.0317626 | -0.8537936 | 3.694953506 | down | 0.001239353 | interferon regulatory factor 7 |
| A_24_P379820 | 1.1778126 | -1.180127 | 5.126377509 | down | 2.21E-04 | integral membrane protein 2C |
| A_24_P380679 | -0.9623138 | 0.74038696 | 3.2550974 | up | 0.00908254 | chromosome 7 open reading frame 53 |
| A_24_P389916 | 1.2645862 | -1.4098475 | 6.38388116 | down | 0.002284279 | leucine rich repeat containing 32 |
| A_24_P392110 | -2.1834173 | 1.9416106 | 17.448463 | up | 5.26E-04 | pregnancy specific beta-1-glycoprotein 8 |
| A_24_P402080 | 0.7894664 | -0.7964473 | 3.001978544 | down | 3.34E-04 | myelin basic protein |
| A_24_P402690 | 1.2035878 | -1.1015317 | 4.942083965 | down | 6.28E-04 | integral membrane protein 2C |
| A_24_P40626 | 1.2769852 | -1.0989531 | 5.190733503 | down | 0.001806182 | gremlin 2, cysteine knot superfamily, homolog (Xenopus laevis) |
| A_24_P408047 | 1.3456733 | -1.1409464 | 5.604632206 | down | 0.00160542 | pleckstrin homology domain containing, family A (phosphoinositide binding specific) member 4 |
| A_24_P412156 | 1.5233425 | -1.44469 | 7.824685113 | down | 5.38E-04 | chemokine (C-X-C motif) ligand 12 (stromal cell-derived factor 1) |
| A_24_P44462 | -0.8498297 | 0.86152554 | 3.274683 | up | 7.99E-04 | tropomyosin 1 (alpha) |
| A_24_P48204 | 1.1800833 | -1.044719 | 4.674468386 | down | 6.79E-04 | secreted and transmembrane 1 |
| A_24_P48723 | 1.5675235 | -1.6803663 | 9.499751178 | down | 0.001394367 | prostaglandin I2 (prostacyclin) synthase |
| A_24_P55496 | 1.2196035 | -1.1551508 | 5.18647558 | down | 5.53E-05 | odd-skipped related 2 (Drosophila) |
| A_24_P557479 | 0.968078 | -1.0979617 | 4.187355869 | down | 0.005409035 | XIAP associated factor 1 |
| A_24_P64167 | 0.84405494 | -0.7785797 | 3.079368854 | down | 0.001025523 | prostaglandin-endoperoxide synthase 1 (prostaglandin G/H synthase and cyclooxygenase) |
| A_24_P678104 | 1.0778034 | -1.0560083 | 4.388754851 | down | 3.02E-05 | stathmin-like 3 |
| A_24_P68908 | -1.1992996 | 0.8248998 | 4.0676613 | up | 0.005157836 | similar to hCG2041270 |
| A_24_P691826 | -2.2245579 | 1.9423814 | 17.962786 | up | 0.002049689 |  |
| A_24_P71244 | -1.2441118 | 1.2488314 | 5.6292524 | up | 4.45E-05 | phosphoinositide-3-kinase, catalytic, delta polypeptide |
| A_24_P80204 | 2.4624875 | -2.5203483 | 31.62154325 | down | 7.20E-05 | mal, T-cell differentiation protein-like |
| A_24_P827037 | -1.1370257 | 1.1319203 | 4.8197093 | up | 7.46E-05 | leucine rich repeat containing 15 |
| A_24_P870620 | 1.0539503 | -0.9355626 | 3.971028961 | down | 6.18E-04 | pleiotrophin |
| A_24_P931443 | -1.6266097 | 1.5815248 | 9.241549 | up | 5.37E-04 | G protein-coupled receptor 68 |
| A_24_P941787 | -0.95539594 | 0.7644088 | 3.2939184 | up | 0.006261525 | PRP4 pre-mRNA processing factor 4 homolog B (yeast) |
| A_24_P942068 | -1.1228461 | 1.1060293 | 4.687684 | up | 3.02E-05 | tetratricopeptide repeat, ankyrin repeat and coiled-coil containing 2 |
| A_24_P945059 | -1.2582928 | 0.9171548 | 4.5172586 | up | 0.003389232 | myc target 1 |
| A_24_P945113 | 0.88937473 | -0.9584875 | 3.59966382 | down | 3.28E-04 | activin A receptor type II-like 1 |
| A_32_P108254 | 2.78185 | -2.850159 | 49.59108924 | down | 1.67E-04 | family with sequence similarity 20, member A |
| A_32_P108655 | -1.0342747 | 1.0180476 | 4.1477313 | up | 0.001264679 | adenylate kinase 3-like 1 |
| A_32_P112493 | 1.1854408 | -0.97534037 | 4.471568982 | down | 0.001832067 | protein kinase-like protein SgK493 |
| A_32_P114284 | -0.94477785 | 0.75671816 | 3.2523804 | up | 0.0063214 | IKAROS family zinc finger 2 (Helios) |
| A_32_P119033 | 2.2036679 | -2.057005 | 19.16859405 | down | 1.80E-04 | phosphatidylinositol-specific phospholipase C, X domain containing 3 |
| A_32_P125338 | 1.4264559 | -1.5263147 | 7.74234408 | down | 2.16E-04 | family with sequence similarity 43, member B |
| A_32_P142440 | 1.9371437 | -1.7607801 | 12.97735076 | down | 5.92E-04 | proprotein convertase subtilisin/kexin type 9 |
| A_32_P157945 | -1.0215067 | 0.9051378 | 3.8016994 | up | 4.80E-04 | desmoplakin |
| A_32_P164246 | -1.9215448 | 1.7253287 | 12.52617 | up | 0.001003558 | forkhead box Q1 |
| A_32_P164593 | 1.3752155 | -1.3252777 | 6.500241419 | down | 1.36E-04 | zinc finger, matrin type 4 |
| A_32_P192474 | 1.0861924 | -0.78389263 | 3.655541271 | down | 0.006578423 | proline-rich transmembrane protein 1 |
| A_32_P196142 | -0.9567447 | 0.6891613 | 3.1294432 | up | 0.008069932 | hypothetical LOC100130938 |
| A_32_P198731 | 1.7063019 | -1.721049 | 10.7580973 | down | 3.69E-04 | neuralized homolog 1B (Drosophila) |
| A_32_P313405 | -0.8757596 | 1.0046077 | 3.6816878 | up | 4.62E-04 | laminin, alpha 1 |
| A_32_P32413 | 1.2711802 | -1.1407217 | 5.321754614 | down | 0.001040802 | SET binding protein 1 |
| A_32_P34444 | -1.7284204 | 1.7045002 | 10.799708 | up | 5.53E-05 | formin homology 2 domain containing 3 |
| A_32_P358887 | -1.6604047 | 1.6271293 | 9.764418 | up | 3.02E-05 | solute carrier family 4, sodium bicarbonate cotransporter, member 4 |
| A_32_P377880 | -1.4113393 | 1.3772795 | 6.9096794 | up | 4.45E-05 | glial cell derived neurotrophic factor |
| A_32_P37867 | 0.9643431 | -1.0529866 | 4.048337965 | down | 2.88E-04 | KIAA1644 |
| A_32_P54553 | 0.9205463 | -0.94063354 | 3.633046335 | down | 0.001192563 | ubiquitin specific peptidase 41 |
| A_32_P57810 | 1.0434375 | -1.0341905 | 4.221126587 | down | 6.02E-05 | ring finger protein 157 |
| A_32_P68504 | -1.1583745 | 0.82129765 | 3.9440346 | up | 0.005483806 | zinc finger, DBF-type containing 2 |
| A_32_P70315 | 1.1340034 | -1.1715748 | 4.943655673 | down | 4.32E-04 | TIMP metallopeptidase inhibitor 4 |
| A_32_P83049 | 1.1979315 | -1.3423169 | 5.816891357 | down | 3.07E-04 | EFR3 homolog B (S. cerevisiae) |
| A_32_P85676 | 1.3961856 | -1.4440455 | 7.161348256 | down | 2.85E-05 | serine/threonine kinase 32B |
| A_32_P87013 | -1.6952953 | 1.7310574 | 10.750655 | up | 8.23E-05 | interleukin 8 |
| A_33_P3214159 | -1.2797279 | 1.1812897 | 5.5060496 | up | 7.20E-05 | cadherin 2, type 1, N-cadherin (neuronal) |
| A_33_P3214466 | 0.7313204 | -0.8766407 | 3.048207369 | down | 0.002616131 | mesoderm posterior 1 homolog (mouse) |
| A_33_P3214720 | -1.040953 | 0.96465874 | 4.0155897 | up | 9.94E-04 | zinc finger CCCH-type containing 12A |
| A_33_P3215640 | 1.308459 | -1.2894547 | 6.054105053 | down | 8.96E-05 | peptidase inhibitor 16 |
| A_33_P3216133 | 1.4099443 | -1.676687 | 8.495102022 | down | 0.001762131 | zinc finger, matrin type 4 |
| A_33_P3219596 | 1.2822022 | -1.4220316 | 6.517117078 | down | 0.001356453 | hypothetical LOC254559 |
| A_33_P3220470 | 1.2712812 | -1.3401643 | 6.111156436 | down | 1.55E-04 | SMAD family member 6 |
| A_33_P3220911 | 1.7424119 | -1.8296988 | 11.89357557 | down | 4.45E-05 | bone marrow stromal cell antigen 2 |
| A_33_P3223056 | 1.1275092 | -1.1094732 | 4.71409975 | down | 0.001449709 | ADAM metallopeptidase with thrombospondin type 1 motif, 10 |
| A_33_P3225512 | 1.5209574 | -1.4622353 | 7.907341769 | down | 1.10E-04 | 2'-5'-oligoadenylate synthetase 2, 69/71kDa |
| A_33_P3225522 | 1.0051141 | -1.0878229 | 4.266156821 | down | 3.34E-04 | 2'-5'-oligoadenylate synthetase 2, 69/71kDa |
| A_33_P3226212 | -0.9429573 | 0.8452189 | 3.45378 | up | 1.69E-04 | junctional adhesion molecule 2 |
| A_33_P3227400 | 1.0914024 | -1.1364385 | 4.684324101 | down | 0.005520597 | collagen, type IV, alpha 4 |
| A_33_P3227793 | 0.90481544 | -0.99437296 | 3.730032901 | down | 0.001296626 | cell growth regulator with EF-hand domain 1 |
| A_33_P3229122 | 0.8588176 | -0.88860476 | 3.357581338 | down | 0.03428638 | histone cluster 1, H2bf |
| A_33_P3237150 | -1.2013388 | 0.95387554 | 4.454348 | up | 0.002143235 | bone morphogenetic protein 2 |
| A_33_P3238215 | -0.99824107 | 0.70773387 | 3.2624934 | up | 0.003807884 | COBL-like 1 |
| A_33_P3238290 | -1.931422 | 1.3137882 | 9.482123 | up | 0.0060587 | family with sequence similarity 65, member C |
| A_33_P3241269 | -2.6884027 | 2.5737507 | 38.376564 | up | 1.22E-04 | carboxylesterase 1 (monocyte/macrophage serine esterase 1) |
| A_33_P3241511 | -1.0386833 | 0.9378655 | 3.9355052 | up | 0.006548095 | serpin peptidase inhibitor, clade D (heparin cofactor), member 1 |
| A_33_P3242733 | 1.2174909 | -0.9516165 | 4.4974508 | down | 0.001723847 | protein kinase-like protein SgK493 |
| A_33_P3242863 | 0.7953346 | -0.839877 | 3.106330951 | down | 3.99E-04 | 5',3'-nucleotidase, mitochondrial |
| A_33_P3243093 | -0.8348428 | 1.0341091 | 3.6526713 | up | 5.89E-04 | regulator of G-protein signaling 5 |
| A_33_P3243230 | -1.3749105 | 1.0904353 | 5.522593 | up | 0.001917958 |  |
| A_33_P3243449 | 1.5684624 | -1.4472518 | 8.087614094 | down | 4.15E-04 | CD70 molecule |
| A_33_P3243887 | -1.565913 | 1.6008296 | 8.980168 | up | 6.14E-04 | interleukin 11 |
| A_33_P3245178 | -0.91892076 | 0.67814755 | 3.0252793 | up | 0.004358128 | brain expressed X-linked 2 |
| A_33_P3246833 | -1.635591 | 1.5734358 | 9.247266 | up | 0.001202556 | interleukin 1 receptor antagonist |
| A_33_P3248354 | 1.5064688 | -1.7168065 | 9.339046277 | down | 0.00232364 | olfactomedin 1 |
| A_33_P3248439 | 1.3939798 | -1.4507866 | 7.18389566 | down | 7.82E-04 | family with sequence similarity 125, member B |
| A_33_P3248982 | 1.2329144 | -1.173343 | 5.300973646 | down | 1.33E-04 | Fas apoptotic inhibitory molecule 2 |
| A_33_P3249534 | -1.4763739 | 1.4049437 | 7.3682275 | up | 2.16E-04 | neurofilament, medium polypeptide |
| A_33_P3249872 | 1.1246264 | -1.0354857 | 4.469495869 | down | 8.81E-05 | fibulin 1 |
| A_33_P3249976 | -1.2369808 | 0.42593288 | 3.1665542 | up | 0.03777026 | junctional adhesion molecule 2 |
| A_33_P3251703 | 1.3947625 | -1.4910293 | 7.391113561 | down | 1.79E-04 | cysteine-rich protein 1 (intestinal) |
| A_33_P3252286 | 0.8654027 | -0.7881341 | 3.146039632 | down | 2.53E-04 | cytokine receptor-like factor 1 |
| A_33_P3252695 | 1.2032232 | -1.0229752 | 4.678994319 | down | 0.001994861 | cytokine-like 1 |
| A_33_P3252781 | 1.0312712 | -1.1085277 | 4.407005852 | down | 6.52E-04 | placenta-specific 9 |
| A_33_P3252785 | 1.1353874 | -1.098429 | 4.703766212 | down | 4.38E-04 |  |
| A_33_P3255304 | 1.1718163 | -1.1840298 | 5.118943778 | down | 0.001229483 | gamma-glutamyltransferase 5 |
| A_33_P3257678 | 1.2382133 | -1.1057124 | 5.076822478 | down | 0.008172127 | histone cluster 2, H3a |
| A_33_P3258617 | -0.9217051 | 0.9308617 | 3.6114216 | up | 8.03E-04 |  |
| A_33_P3262635 | 0.9212756 | -0.8699992 | 3.461205853 | down | 7.88E-04 | cat eye syndrome chromosome region, candidate 1 |
| A_33_P3265030 | 1.0255926 | -1.0112963 | 4.103596273 | down | 4.57E-04 | septin 5 |
| A_33_P3266025 | -1.7033689 | 1.222573 | 7.599697 | up | 0.00401423 | similar to mitochondrial cytochrome c oxidase subunit Vb |
| A_33_P3268892 | -1.0242467 | 1.168916 | 4.573069 | up | 6.28E-04 |  |
| A_33_P3268910 | 0.9727578 | -1.0182614 | 3.975177402 | down | 1.91E-04 |  |
| A_33_P3269636 | 0.9694741 | -1.0138996 | 3.954166466 | down | 8.68E-05 | suprabasin |
| A_33_P3270311 | -0.95649934 | 0.8796389 | 3.57053 | up | 3.28E-04 | HECT, C2 and WW domain containing E3 ubiquitin protein ligase 2 |
| A_33_P3270776 | 1.3524759 | -1.3326312 | 6.431286304 | down | 6.11E-05 | HtrA serine peptidase 3 |
| A_33_P3271635 | 1.8182515 | -1.6141601 | 10.79589963 | down | 1.53E-04 | major histocompatibility complex, class II, DP beta 1 |
| A_33_P3271651 | 1.5511298 | -1.4025149 | 7.747038133 | down | 1.79E-04 | major histocompatibility complex, class II, DP beta 1 |
| A_33_P3275500 | -1.1163945 | 0.78966534 | 3.7478414 | up | 0.007205636 | FLJ45933 protein |
| A_33_P3276713 | 1.4618647 | -1.3809679 | 7.174272771 | down | 7.20E-05 | hepatocyte growth factor (hepapoietin A; scatter factor) |
| A_33_P3276718 | 0.78284836 | -0.8373735 | 3.074223152 | down | 1.39E-04 | hepatocyte growth factor (hepapoietin A; scatter factor) |
| A_33_P3280521 | -1.458609 | 1.231132 | 6.4519763 | up | 0.001103588 | microfibrillar-associated protein 3-like |
| A_33_P3280845 | 0.9470253 | -0.8685832 | 3.520080652 | down | 5.64E-04 | Thy-1 cell surface antigen |
| A_33_P3280993 | -0.88032544 | 0.78192556 | 3.1650999 | up | 0.010995622 | HLA complex group 18 |
| A_33_P3283601 | 1.0713615 | -0.95984626 | 4.087469058 | down | 3.34E-04 | placenta-specific 9 pseudogene |
| A_33_P3284404 | 1.106215 | -1.105061 | 4.630846942 | down | 2.12E-04 | synaptogyrin 1 |
| A_33_P3285545 | 0.9645872 | -0.82012224 | 3.445490717 | down | 6.46E-04 | claudin 4 |
| A_33_P3287959 | 0.79765356 | -0.8229319 | 3.074997844 | down | 2.35E-04 | RAS p21 protein activator 4 |
| A_33_P3290707 | -0.92190075 | 0.77657485 | 3.2455783 | up | 0.001132522 | membrane metallo-endopeptidase |
| A_33_P3296497 | -0.9903923 | 0.6669135 | 3.1542692 | up | 0.013272238 |  |
| A_33_P3298024 | -1.3879558 | 1.1037619 | 5.6244726 | up | 0.002673081 | ATP-binding cassette, sub-family C (CFTR/MRP), member 3 |
| A_33_P3298159 | 0.98157024 | -0.71390533 | 3.238836395 | down | 0.002099778 | prostaglandin D2 synthase 21kDa (brain) |
| A_33_P3298930 | -0.90903497 | 0.7324934 | 3.1199617 | up | 0.017747339 | similar to hCG1654959 |
| A_33_P3301876 | -1.26624 | 0.92221904 | 4.558183 | up | 0.006050888 | histone deacetylase 4 |
| A_33_P3302125 | -1.2139733 | 0.96618986 | 4.532048 | up | 0.024595555 | late cornified envelope 2A |
| A_33_P3302245 | 0.73337555 | -0.853848 | 3.004705399 | down | 0.006773613 | transmembrane protein 59-like |
| A_33_P3302632 | 1.1333268 | -0.96722794 | 4.288742689 | down | 0.005811628 | histone cluster 1, H2be |
| A_33_P3304212 | 1.2294074 | -1.2436887 | 5.552341059 | down | 5.28E-05 | pleckstrin homology domain containing, family G (with RhoGef domain) member 3 |
| A_33_P3305105 | 1.0869836 | -0.978552 | 4.185893172 | down | 4.32E-04 | von Willebrand factor A domain containing 5A |
| A_33_P3312658 | -1.8360565 | 1.4886837 | 10.019511 | up | 0.001863304 |  |
| A_33_P3315779 | 0.8736584 | -0.75847626 | 3.099713122 | down | 0.002131001 | hect domain and RLD 6 |
| A_33_P3316273 | -2.0083365 | 1.8031902 | 14.040543 | up | 0.001809339 | chemokine (C-C motif) ligand 3 |
| A_33_P3316539 | -1.3055232 | 1.0983088 | 5.2920694 | up | 6.93E-04 | solute carrier family 7 (cationic amino acid transporter, y+ system), member 2 |
| A_33_P3318581 | -1.0518196 | 1.0303297 | 4.2343755 | up | 1.72E-04 | procollagen-lysine, 2-oxoglutarate 5-dioxygenase 2 |
| A_33_P3320538 | -1.0286744 | 0.77937603 | 3.5016875 | up | 0.007091114 |  |
| A_33_P3325704 | -1.5926542 | 1.5796461 | 9.014831 | up | 1.38E-04 | small proline-rich protein 2E |
| A_33_P3326588 | -1.0839813 | 0.8323147 | 3.7745273 | up | 0.003603906 | tumor necrosis factor receptor superfamily, member 10d, decoy with truncated death domain |
| A_33_P3329078 | 1.8115995 | -1.4728763 | 9.743741546 | down | 0.002377953 | hemoglobin, gamma A |
| A_33_P3329686 | 0.924122 | -1.0437912 | 3.912018699 | down | 0.003603906 | septin 5 |
| A_33_P3330264 | -0.9327204 | 0.885607 | 3.526721 | up | 8.60E-04 | chemokine (C-X-C motif) ligand 1 (melanoma growth stimulating activity, alpha) |
| A_33_P3332081 | -1.8033634 | 1.7212648 | 11.508504 | up | 5.01E-04 | KH domain containing, RNA binding, signal transduction associated 3 |
| A_33_P3341499 | -0.9351425 | 1.0443587 | 3.9435673 | up | 8.51E-04 | wingless-type MMTV integration site family, member 5A |
| A_33_P3342081 | -1.7744514 | 1.4787502 | 9.534794 | up | 0.001590924 | PR domain containing 1, with ZNF domain |
| A_33_P3342917 | -0.97428167 | 0.9691849 | 3.8462873 | up | 3.21E-04 | synaptogyrin 2 |
| A_33_P3347291 | 0.8842974 | -0.805032 | 3.225067599 | down | 0.001009482 | indolethylamine N-methyltransferase |
| A_33_P3347869 | -2.8510718 | 2.2457356 | 34.22094 | up | 0.002095723 | complement component 3 |
| A_33_P3348011 | -1.1754786 | 0.6934755 | 3.6526766 | up | 0.013220662 | transducer of ERBB2, 2 pseudogene |
| A_33_P3350726 | 1.0473847 | -1.2600044 | 4.949865289 | down | 7.24E-04 | peroxisome proliferator-activated receptor gamma |
| A_33_P3351536 | 0.8937571 | -1.0759734 | 3.916949239 | down | 0.006703007 | PTK2B protein tyrosine kinase 2 beta |
| A_33_P3351999 | -0.9766133 | 0.97284937 | 3.8623066 | up | 7.46E-05 |  |
| A_33_P3352970 | -0.95240855 | 0.9884722 | 3.8393998 | up | 2.16E-04 | interleukin-1 receptor-associated kinase 2 |
| A_33_P3353791 | -1.3371141 | 0.93741393 | 4.838393 | up | 0.013922806 | integrin, alpha 1 |
| A_33_P3357530 | 0.968261 | -0.9280224 | 3.722529639 | down | 5.53E-05 | solute carrier family 12 (potassium/chloride transporters), member 7 |
| A_33_P3360611 | 0.8618028 | -0.81193185 | 3.190394106 | down | 1.68E-04 | membrane protein, palmitoylated 2 (MAGUK p55 subfamily member 2) |
| A_33_P3362008 | -2.076064 | 1.8281782 | 14.97249 | up | 3.48E-04 | natriuretic peptide precursor B |
| A_33_P3364864 | -1.0714716 | 0.99492073 | 4.1883802 | up | 6.48E-04 | nicotinamide phosphoribosyltransferase |
| A_33_P3364869 | -1.5581995 | 1.0446255 | 6.0747504 | up | 0.009658951 | nicotinamide phosphoribosyltransferase |
| A_33_P3369844 | -1.0074241 | 0.9501262 | 3.8840191 | up | 8.03E-04 | CD24 molecule |
| A_33_P3370094 | -0.98945284 | 0.8110366 | 3.4833837 | up | 0.00230271 | membrane metallo-endopeptidase |
| A_33_P3371718 | -0.88610077 | 0.8813791 | 3.4045873 | up | 2.16E-04 | spermidine/spermine N1-acetyltransferase 1 |
| A_33_P3375934 | -1.136586 | 0.94721055 | 4.239213 | up | 0.001316656 | nicotinamide phosphoribosyltransferase |
| A_33_P3376140 | -1.6585754 | 1.2864639 | 7.7009654 | up | 0.017888622 | potassium inwardly-rectifying channel, subfamily J, member 15 |
| A_33_P3379091 | 0.80558205 | -0.98491454 | 3.459339785 | down | 0.002672263 | synaptogyrin 1 |
| A_33_P3379886 | -1.596421 | 1.0222149 | 6.1416907 | up | 0.004713418 | fibroblast growth factor 2 (basic) |
| A_33_P3381338 | 1.1042354 | -1.0478117 | 4.44458035 | down | 5.01E-04 | tenascin XB |
| A_33_P3382856 | 1.6728835 | -1.2329383 | 7.494445679 | down | 0.003547048 | decorin |
| A_33_P3384287 | 2.0385697 | -1.9958379 | 16.38617898 | down | 2.35E-05 | paralemmin |
| A_33_P3393135 | 0.8962575 | -0.7629783 | 3.158491858 | down | 0.009183117 |  |
| A_33_P3393821 | 1.2569795 | -0.9830487 | 4.72406277 | down | 0.003011333 | complement component 1, r subcomponent |
| A_33_P3394727 | -1.819315 | 1.6282737 | 10.910071 | up | 4.62E-04 | KH domain containing, RNA binding, signal transduction associated 3 |
| A_33_P3395605 | 1.1464863 | -0.98203254 | 4.37268327 | down | 6.52E-04 | transmembrane protein 119 |
| A_33_P3396527 | -0.90719473 | 0.7295804 | 3.1096995 | up | 0.003806428 | polymerase (RNA) III (DNA directed) polypeptide G (32kD) |
| A_33_P3397763 | 1.2687835 | -1.0514137 | 4.994004697 | down | 9.96E-04 | tumor necrosis factor (ligand) superfamily, member 9 |
| A_33_P3401647 | 0.79246974 | -0.8161149 | 3.049525204 | down | 0.005306378 | protein phosphatase 1, regulatory (inhibitor) subunit 14A |
| A_33_P3401826 | 1.44673 | -1.5789143 | 8.143475 | down | 9.97E-05 | cytidine monophosphate (UMP-CMP) kinase 2, mitochondrial |
| A_33_P3404480 | -0.9639337 | 0.8899648 | 3.6147566 | up | 3.80E-04 | membrane protein, palmitoylated 4 (MAGUK p55 subfamily member 4) |
| A_33_P3406493 | 1.1714735 | -1.1870008 | 5.12827745 | down | 5.28E-05 | gamma-aminobutyric acid (GABA) B receptor, 2 |
| A_33_P3407529 | 1.7279105 | -1.9800355 | 13.06781536 | down | 8.26E-04 | proline-rich transmembrane protein 4 |
| A_33_P3408953 | -0.9262401 | 0.7587609 | 3.2154062 | up | 0.0029287 | gamma-glutamyltransferase light chain 2 |
| A_33_P3409210 | 1.1668302 | -0.9987732 | 4.486540356 | down | 2.77E-04 |  |
| A_33_P3409477 | -0.8451568 | 0.9020066 | 3.3569787 | up | 1.96E-04 | ubiquitin associated and SH3 domain containing, B |
| A_33_P3413987 | 0.94564426 | -0.98902094 | 3.822894036 | down | 2.21E-04 | serpin peptidase inhibitor, clade G (C1 inhibitor), member 1 |
| A_33_P3415340 | -1.0569726 | 0.85920703 | 3.774223 | up | 0.001761928 |  |
| A_33_P3416473 | -1.0585933 | 0.94567394 | 4.011849 | up | 3.37E-04 | hypothetical LOC646999 |
| A_33_P3419190 | -1.1598163 | 1.1689951 | 5.023913 | up | 0.002150099 | amphiregulin |
| A_33_P3419696 | -1.3860646 | 1.0485809 | 5.4063153 | up | 0.00347395 | fibroblast growth factor 2 (basic) |
| A_33_P3421243 | -0.9722979 | 0.89407754 | 3.646154 | up | 0.001132165 | alpha-fetoprotein |
| A_33_P3423551 | -1.408222 | 1.3598528 | 6.8119826 | up | 0.00187072 | immediate early response 3 |
| A_33_P3423941 | 2.3382368 | -2.3175225 | 25.20712061 | down | 5.32E-05 | interferon induced transmembrane protein 1 (9-27) |
| A_33_P3441021 | -1.2326658 | 1.2706738 | 5.669964 | up | 8.17E-05 | transmembrane protein 233 |
| A_33_P3695899 | -1.303551 | 1.0751868 | 5.2008157 | up | 0.001985931 | hypothetical gene supported by AK055666; BC039324 |
| A_33_P3712341 | 1.4256968 | -1.3886632 | 7.034072696 | down | 3.88E-05 | chemokine (C-X-C motif) ligand 12 (stromal cell-derived factor 1) |
| A_33_P3718269 | -1.8618915 | 1.8415062 | 13.026682 | up | 2.21E-04 | hypothetical protein LOC285628 |
| A_33_P3775848 | 0.725693 | -1.0922316 | 3.525736288 | down | 0.005210312 | chloride intracellular channel 2 |
| A_33_P3813128 | -1.0839242 | 1.1818702 | 4.809191 | up | 1.55E-04 | sema domain, immunoglobulin domain (Ig), short basic domain, secreted, (semaphorin) 3F |
| A_33_P3825869 | 1.5376301 | -1.5242189 | 8.350422055 | down | 3.02E-05 | calcium channel, voltage-dependent, L type, alpha 1C subunit |
| A_33_P3835524 | -1.0043668 | 1.0911775 | 4.273874 | up | 0.00102514 | POU class 2 homeobox 2 |
| A_33_P3846177 | 1.3600705 | -1.404554 | 6.795711335 | down | 1.19E-04 | beta-1,4-N-acetyl-galactosaminyl transferase 1 |
| A_33_P3846653 | -1.6230434 | 1.7695615 | 10.502092 | up | 6.10E-04 |  |
| A_33_P3871347 | 1.0686228 | -1.0133078 | 4.233734131 | down | 1.21E-04 | sushi, nidogen and EGF-like domains 1 |
|  |  |  |  |  |  |  |
